# Genome-wide characterization of *SOS1* gene family in potato (*Solanum tuberosum*) and expression analyses under salt and hormone stress

**DOI:** 10.3389/fpls.2023.1201730

**Published:** 2023-06-30

**Authors:** Liqin Liang, Liuyan Guo, Yifan Zhai, Zhiling Hou, Wenjing Wu, Xinyue Zhang, Yue Wu, Xiaona Liu, Shan Guo, Gang Gao, Weizhong Liu

**Affiliations:** College of Life Science, Shanxi Normal University, Taiyuan, China

**Keywords:** *Solanum tuberosum* L., *SOS1*, expression profiles, abiotic stress, genome-wide

## Abstract

Salt Overly Sensitive 1 (SOS1) is one of the members of the Salt Overly Sensitive (SOS) signaling pathway and plays critical salt tolerance determinant in plants, while the characterization of the *SOS1* family in potato (*Solanum tuberosum*) is lacking. In this study, 37 *StSOS1s* were identified and found to be unevenly distributed across 10 chromosomes, with most of them located on the plasma membrane. Promoter analysis revealed that the majority of these *StSOS1* genes contain abundant *cis*-elements involved in various abiotic stress responses. Tissue specific expression showed that 21 of the 37 *StSOS1s* were widely expressed in various tissues or organs of the potato. Molecular interaction network analysis suggests that 25 StSOS1s may interact with other proteins involved in potassium ion transmembrane transport, response to salt stress, and cellular processes. In addition, collinearity analysis showed that 17, 8, 1 and 5 of orthologous *StSOS1* genes were paired with those in tomato, pepper, tobacco, and Arabidopsis, respectively. Furthermore, RT-qPCR results revealed that the expression of *StSOS1s* were significant modulated by various abiotic stresses, in particular salt and abscisic acid stress. Furthermore, subcellular localization in *Nicotiana benthamiana* suggested that StSOS1-13 was located on the plasma membrane. These results extend the comprehensive overview of the *StSOS1* gene family and set the stage for further analysis of the function of genes in SOS and hormone signaling pathways.

## 1 Introduction

High soil salinity is a major abiotic stress that significantly affects plant growth and ultimately reduces plant productivity by preventing the absorption of water and nutrients (Brindha et al., 2021; You et al., 2022). The Salt Overly Sensitive (SOS) signaling pathway plays an essential role in the response of plants to salt stress. It consists of three components: *SOS1*, *SOS2*, and *SOS3* (Cheng et al., 2019). *SOS1* is a Na^+^/H^+^ antitransporter that governs the efflux of Na^+^ into the root and loading into the xylem vessel for long-distance transport out of the root (Świeżawska et al., 2018). *SOS2* exists as a form of protein kinase in the SOS signaling pathway, which in turn activates *SOS1* to bring about sodium ion homeostasis and salt tolerance (Ali et al., 2021). *SOS3*, which encodes an EF-handed Ca^2+^ binding protein, can sense calcium signals elicited by salt stress, interact with SOS2, and activate SOS2 (Zhu et al., 2021).

*SOS1* genes were firstly identified in Arabidopsis (Keisham et al., 2018) and designated as *AtNHX1*-*AtNHX8*. *AtNHX7* (or *AtSOS1*) is a critical player in the SOS signaling pathway (Zhao C. et al., 2021). *AtSOS1* locates in the plasma membrane (Shi et al., 2000). *AtSOS1* is primarily expressed in epidermal cells at the root tip and in the parenchyma at the xylem-symplast boundary of root, stem, and leaf, hinting at the role of this transporter in the extrusion of Na^+^ into the growing medium and in controlling long-distance Na^+^ transport in plants (Gao et al., 2016). *SOS1* behaves as a homodimer, with each monomer having 12 transmembrane domains at its N-terminal region and a long C-terminal region containing a cytosolic domain, a cyclic nucleotide binding domain, and an auto-inhibitory domain (Wu et al., 1996; Núñez-Ramírez et al., 2012). SOS proteins were involved in the regulation of plant tolerance to salinity (Zhu et al., 1998). Overexpression of *SOS1* led to reduction of Na^+^ accumulation in the xylem and shoot (Shi et al., 2003).

In addition to Arabidopsis, the physiological roles of the associated *SOS1* genes have been investigated in cash crop plants, such as soybean, maize, tomato, cotton (Chen et al., 2017; Wang Z. et al., 2021; Zhang M. et al., 2022; Zhou et al., 2022), and so on. In soybeans, significant accumulation of Na^+^ in the roots of *GmSOS1* mutants resulted in an imbalance of Na^+^ and K^+^, suggesting that *GmSOS1* played a critical role in soybean salt tolerance by maintaining Na^+^ homeostasis (Zhang et al., 2022). In maize, SOS pathway has a conserved salt tolerant effect, and its components (*ZmSOS1* and *ZmCBL8*) have Na^+^ regulation and natural variations of salt tolerance, providing an important gene target for breeding salt-tolerant maize (Zhou et al., 2022). However, its role has not yet been investigated in potato (*Solanum tuberosum*).

Potato is an important crop in human food systems around the world (Dahal et al., 2019; Ceci et al., 2022) and their cultivation and production are often severely threatened by the various environmental stresses such as salinity and pathogens (Li et al., 2021; Yang et al., 2022). Identification and characterization of resistance genes to salt stress would therefore be helpful in improving potato production. Since the role of *SOS1* in controlling ion homeostasis has been shown in several plants, this gene family is thought to also be valuable in the salt tolerance mechanism and quality improvement of potato. However, limited efforts have been made to identify gene families in the potato, and their expression patterns and regulatory mechanisms remain unclear.

In this study, we identified and analyzed the *SOS1* gene family in potato. Extensive analysis including chromosomal localization, gene structure, and upstream promoter *cis*-acting elements of these gene family were conducted. The physicochemical properties, motifs, gene ontologies, and phylogenetic relationships between the encoded proteins were predicted using bioinformatics tools. Furthermore, the expression profiles of specific *StSOS1s* at salt stress were examined using RT-qPCR. In addition, their expression profiles in response to the exogenous phytohormone abscisic acid (ABA), methyl jasmonate (MeJA), gibberellin (GA) and salicylic acid (SA) were also investigated. The results indicate a diverse pattern of responses to abiotic stress *via* SOS and hormone signaling pathways. It may be beneficial to elucidate the resistance of the potato to abiotic stress, providing some theoretical basis for molecular breeding.

## 2 Materials and methods

### 2.1 Plant material and treatments

The potato (diploid cultivar *Solanum phureja*, DM1-3 516 R44) plants used in this study were obtained from Institute of Vegetable and Flowers, Chinese Academy of Agricultural Sciences (CAAS). The potato was grown in a growth chamber at 26 °C/18 °C (day/night) with a 16:8 light: dark cycle and 60-70% relative humidity according to (Ali et al., 2014). The roots of 7-8-leaves-old plantlets were watered with 200 mM NaCl solution (Ma et al., 2021). And the leaves were sprayed with 100 μM ABA, 50 μM MeJA, 350 μM GA and 50 μM SA, respectively. When spraying, moisten the positive and negative sides of all leaves with condensed water droplets without dropping. After the spraying, the plants were immediately wrapped in black plastic bags and treated only once (Yu et al., 2021). Then, the 1, 2, 3, 4 and 5 d (0 d as control) treated plant leaves were respectively quickly frozen in liquid nitrogen at -80 °C for later use (Li et al., 2021). And each treatment was repeated three times.

### 2.2 *SOS1* genes identification in the potato

All protein sequences were obtained from potato genome data (SolTub_3.0)^1^. First, the HMM profile for the SOS1s domain (PF00999) was downloaded from the Pfam server^2^. Then, the HMMER program^3^ was used to identify the SOS1 proteins in the potato genome (Liang et al., 2017). Finally, the SOS1 (Na^+^/H^+^ exchanger, NHX) domain of all putative SOS1 proteins were determined through CDD^4^ and SMART databases^5^. A total of 37 putative *SOS1* genes were identified.

### 2.3 Biophysical properties and chromosomal location analysis

Biophysical characteristics of SOS1 proteins were analyzed through ExPASy webserver^6^ (Wang T. et al., 2021) and NetPhos 3.1^7^ (Naureen et al., 2023). The online prediction tool UniProt^8^ (Ilzhöfer et al., 2022) was applied to predict the tertiary structures of potato SOS1s. Subcellular location of protein was predicted using the Cell-PLoc 2.0 prediction tool^9^. The physical positions of the *StSOS1s* along each chromosome were identified from the potato genome database and the distribution of *StSOS1s* was plotted (Xiang et al., 2016).

### 2.4 *StSOS1s cis*-acting element analysis

The 2000 bp upstream region of the ATG start codon was submitted to PlantCARE^10^ (Koul et al., 2019) to identify the *cis*-acting elements and calculate the number of each element. These promoter sequences were represented as word clouds with the help of the WordArt tool^11^ (Sharma et al., 2021).

### 2.5 Conserved motifs and gene structure analysis

The conserved motifs in StSOS1s were identified to use the MEME website^12^ (Multiple Em for Motif Elicitation) (Zhang et al., 2021) with the maximum number of motifs was set to 10. Figures of phylogenetic tree along with gene conserved motifs and CDS/UTR structure of StSOS1s were drawn with TBtools (v1.098) (Chen et al., 2020) software. Gene Structure Display Server (GSDS)^13^ (Sun et al., 2022) and MEME webserver were employed for gene structure analysis.

### 2.6 *StSOS1s* tissue-specific expressions and GO enrichment

RNA-Seq data (fragments per kilobase of exon per million mapped, FPKM) (NCBI accession number ERP000527) in potato DM genotype (Wang J. et al., 2021) was used to analyze the expression level of *StSOS1* genes. PlantRegMap^14^ (Li H. et al., 2020) was used to functionally re-annotate the proteome of up or down-regulated genes and to plot gene ontology (GO) annotations. Protein-protein interaction (PPI) enrichment was computed by STRING^15^ (Fayez et al., 2022) tool, in which Cytoscape software was used for reconstructing the PPI network, modules and to detect the relationship between overall targeted genes.

### 2.7 Evolutionary tree construction and collinearity analysis

The SOS1 protein sequences of Arabidopsis, tomato, pepper and tobacco were downloaded from the EnsemblPlants (Contreras-Moreira et al., 2022). Homologous sequences were fed into the MEGA7 software and the Clustalw program was used to perform multi-sequence alignment. The results of the output multi-sequence alignment were used to construct an evolutionary tree using the proximity method (He et al., 2022). The collinearity of the sequences of potato with other four species was extracted using TBtools (Zhang C. et al., 2022).

### 2.8 RNA isolation and RT-qPCR analysis

The leaves samples were ground into powder in liquid nitrogen, total RNA was extracted using *TransZol* Up Plus RNA kit (Trans, Beijing, China), following the manufacturer’s instruction. Then the extracted RNA was employed as a template with *TransScript* ^®^ One-Step gDNA Removal and cDNA Synthesis SuperMix for qPCR (Trans, Beijing, China) for the first strand cDNA synthesis. All primer sequences used in this study were designed by Primer Blast website^16^ of NCBI (**Table S1**). The RT-qPCR was performed on a QuantStudio-3 system (Thermo Fisher Scientific, Shanghai, China). The reaction system was 20 µL (cDNA 1 µL, *SOS1*-F 0.4µL, *SOS1*-R 0.4µL, SuperMix 10 µL, DyeII 0.4µL, Water 7.8 µL). The reaction system was 94 °C 30 s, (94 °C 5 s, 60 °C 30 s) ×40 Cycles. Three replications were performed and the expression values were calculated by using the 2^−ΔΔCT^ method (Mo et al., 2022).

### 2.9 Subcellular localization of StSOS1-13

For the localization and expression of StSOS1-13 in potato, the CDS without the stop codon was cloned into pCAMBIA1300. Firstly, the complete coding region of *StSOS1-13* (1 734bp) was amplified from the cDNA by PCR using a pair of primers with a homologous arm and inserted into the pCAMBIA1300 vector linearized by the restriction enzyme *Nco*I. Then, the obtained pStSOS1-13-GFP fusion plasmid was converted into *Escherichia coli* DH5α for verified by bacterial liquid PCR and company sequencing (Sangon, Shanghai, China), further inserted into individual *Agrobacterium tumefaciens* strain GV3101 cells and a single colony was selected for PCR positive identification. Finally, the expression vectors were injected into tobacco leaves for the transient expression experiments (Luo et al., 2022). GFP expression was analyzed using scanning confocal laser microscopy.

## 3 Results

### 3.1 Identification of *SOS1* genes in the potato

**Figure 1:**
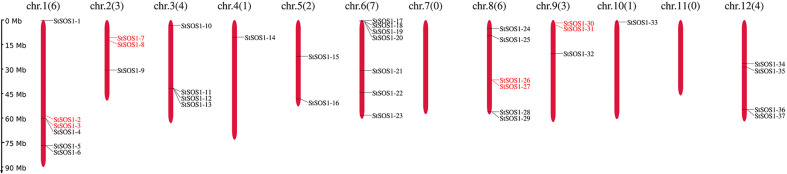
Distribution of the *StSOS1* genes in the potato on 12 chromosomes. The nomenclature for *StSOS1* members was based on the physical position from top to bottom on the chromosome, the names were displayed on the right-hand side of each chromosome, the number of chromosomes and the *StSOS1* genes were indicated at the top of each chromosome, and the scale of the genome size was given on the left-hand side. All protein sequences were obtained from potato genome data (SolTub_3.0).

To identify the *SOS1s* family members in potato, the similar protein sequences were searched in the HMMER program with the query sequence SOS1s motif (PF00999). The SMART tool was then used to confirm whether the candidates contained the Na^+^/H^+^ exchanger (NHX) domain. In total, 37 *SOS1* genes were retrieved from the potato genome and renamed *StSOS1-1* to *StSOS1-37* based on their relative linear order on each chromosome, following the widely used nomenclature (**Figure 1**). Meantime, we found four pairs of tandem duplicated genes existed in 37 *StSOS1* genes. The analysis showed that there was one pair of tandem duplicated genes (*StSOS1-2* and *StSOS1-3*) on Chr1, one pair (*StSOS1-7* and *StSOS1-8*) on Chr2, one pair (*StSOS1-26* and *StSOS1-27*) on Chr6, and one pair (*StSOS1-30* and *StSOS1-31*) on Chr9.

We further determined the biophysical properties of the potato *SOS1* genes including the locus ID, protein length (aa), predicted protein molecular weight (MW), isoelectric points (pI), and NHX domain. The statistical results showed that the protein length ranged from 209 (*StSOS1-15*) to 1153 (*StSOS1-1*) amino acids, the average amino acids length and molecular weights ranged from 22.51 KDa (*StSOS1-15*) to 127.86 KDa (*StSOS1-1*). PI varying from 4.96 (*StSOS1-20*) to 10.12 (*StSOS1-3*). The subcellular localization of these *StSOS1s* predicted through Cell-PLoc 2.0 tool revealed that most of the StSOS1 proteins were localized in the plasma membrane (**Table 1**). The results of the NetPhos 3.1 server revealed that StSOS1 proteins were phosphorylated, and phosphorylated residues were Serine (Ser), threonine (Thr) and tyrosine (Tyr) (**Table S2**), among which serine prediction sites ranged from 11 (StSOS1-24) to 72 (StSOS1-1). The threonine prediction sites ranged from 5 (StSOS1-15) to 32 (StSOS1-1), and the tyrosine prediction sites ranged from 0 (StSOS1-15 and StSOS1-20) to 9 (StSOS1-21). Three-dimensional protein models were constructed by sequence similarity search using UniProt PDB database and the homology modeling was predicted by DS Visualizer (**Figure S1**). The structures of *StSOS1-7*, *StSOS1-8*, *StSOS1-9*, *StSOS1-16*, *StSOS1-17*, *StSOS1-21*, *StSOS1-22*, *StSOS1-25*, *StSOS1-28*, *StSOS1-29*, *StSOS1-31*, *StSOS1-32*, *StSOS1-36*, and *StSOS1-37* are similar and suggest shared functionality, as do *StSOS1-18* and *StSOS1-19*. These provide an initial basis for understanding the molecular function of the StSOS1 proteins.

**Table 1:** Detailed information regarding StSOS1 proteins in the potato.

Gene Name	Gene ID	Transcript ID	AA Number	MW (KDa)	pI	Na^+^/H^+^ Exchanger Domain (start-end)	Localization
*StSOS1-1*	PGSC0003DMG400022786	PGSC0003DMT400058653	1153	127.86	5.87	29-459	Plasma membrane
*StSOS1-2*	PGSC0003DMG400010663	PGSC0003DMT400027658	537	59.45	8.55	21-445	Extracellular
*StSOS1-3*	PGSC0003DMG400010663	PGSC0003DMT400027657	252	28.25	10.12	1-160	Extracellular
*StSOS1-4*	PGSC0003DMG400010663	PGSC0003DMT400027656	478	53.16	7.70	21-430	Extracellular
*StSOS1-5*	PGSC0003DMG400022490	PGSC0003DMT400057914	411	45.49	7.80	8-320	Extracellular
*StSOS1-6*	PGSC0003DMG400022490	PGSC0003DMT400057913	536	58.81	7.70	26-445	Extracellular
*StSOS1-7*	PGSC0003DMG400021928	PGSC0003DMT400056443	694	76.70	5.53	1-685	Plasma membrane
*StSOS1-8*	PGSC0003DMG400021928	PGSC0003DMT400056445	813	89.81	5.69	12-804	Extracellular
*StSOS1-9*	PGSC0003DMG400009710	PGSC0003DMT400025130	823	89.10	8.82	8-783	Plasma membrane
*StSOS1-10*	PGSC0003DMG400018689	PGSC0003DMT400048101	790	87.29	5.97	28-777	Plasma membrane
*StSOS1-11*	PGSC0003DMG400031029	PGSC0003DMT400079669	294	32.09	8.45	1-262	Membrane
*StSOS1-12*	PGSC0003DMG400031029	PGSC0003DMT400079670	500	54.21	8.82	92-464	Plasma membrane
*StSOS1-13*	PGSC0003DMG400031029	PGSC0003DMT400079671	577	62.96	7.14	169-541	Plasma membrane
*StSOS1-14*	PGSC0003DMG400027255	PGSC0003DMT400070102	791	87.48	7.89	43-775	Plasma membrane
*StSOS1-15*	PGSC0003DMG400009808	PGSC0003DMT400025403	209	22.51	4.50	162-204	Plasma membrane
*StSOS1-16*	PGSC0003DMG400011649	PGSC0003DMT400030419	793	87.85	6.74	23-790	Plasma membrane
*StSOS1-17*	PGSC0003DMG400007292	PGSC0003DMT400018809	807	89.34	8.19	24-806	Plasma membrane
*StSOS1-18*	PGSC0003DMG402021988	PGSC0003DMT400056557	269	30.01	8.81	2-179	Extracellular
*StSOS1-19*	PGSC0003DMG402021988	PGSC0003DMT400056556	306	34.27	9.11	3-216	Extracellular
*StSOS1-20*	PGSC0003DMG402021988	PGSC0003DMT400056555	252	27.51	4.96	25-231	Extracellular
*StSOS1-21*	PGSC0003DMG402021988	PGSC0003DMT400061554	832	91.61	7.08	13-773	Plasma membrane
*StSOS1-22*	PGSC0003DMG400013814	PGSC0003DMT400035881	841	91.99	6.61	11-827	Plasma membrane
*StSOS1-23*	PGSC0003DMG400030375	PGSC0003DMT400078102	738	80.33	7.10	1-692	Plasma membrane
*StSOS1-24*	PGSC0003DMG400035252	PGSC0003DMT400085681	424	45.08	9.03	3-408	Plasma membrane
*StSOS1-25*	PGSC0003DMG400030154	PGSC0003DMT400077544	832	91.88	5.37	17-778	Plasma membrane
*StSOS1-26*	PGSC0003DMG400029945	PGSC0003DMT400076994	599	64.77	7.6	179-551	Plasma membrane
*StSOS1-27*	PGSC0003DMG400029945	PGSC0003DMT400076993	389	41.77	5.65	175-367	Plasma membrane
*StSOS1-28*	PGSC0003DMG400012169	PGSC0003DMT400031718	802	87.00	8.64	3-798	Plasma membrane
*StSOS1-29*	PGSC0003DMG400012168	PGSC0003DMT400031717	802	86.63	8.57	3-778	Plasma membrane
*StSOS1-30*	PGSC0003DMG400008849	PGSC0003DMT400022808	679	74.74	9.02	12-672	Plasma membrane
*StSOS1-31*	PGSC0003DMG400008849	PGSC0003DMT400022809	796	87.69	8.71	12-774	Plasma membrane
*StSOS1-32*	PGSC0003DMG400004171	PGSC0003DMT400010686	789	87.81	6.80	14-781	Plasma membrane
*StSOS1-33*	PGSC0003DMG400034953	PGSC0003DMT400085382	548	61.77	7.73	40-490	Extracellular
*StSOS1-34*	PGSC0003DMG400014998	PGSC0003DMT400038811	628	69.31	5.98	26-623	Plasma membrane
*StSOS1-35*	PGSC0003DMG400014998	PGSC0003DMT400038812	777	85.91	8.40	31-772	Plasma membrane
*StSOS1-36*	PGSC0003DMG400005009	PGSC0003DMT400012866	793	86.34	8.16	5-773	Plasma membrane
*StSOS1-37*	PGSC0003DMG400005009	PGSC0003DMT400012865	791	86.13	8.16	5-771	Plasma membrane

* http://plants.ensembl.org/index.html.

### 3.2 Prediction of *cis*-elements in the promoter sequences of *StSOS1* genes

**Figure 2:**
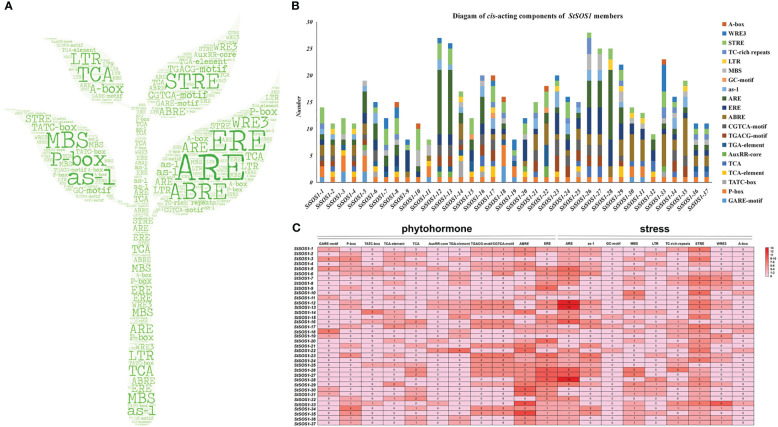
Analysis of the *cis*-acting elements. **(A)** Word clouds representing different *cis*-regulatory elements present at 2000 bp upstream of *StSOS1* genes sequences. **(B)** Graphical representation of 37 *StSOS1* genes with various roles in hormonal response, abiotic stress, plant development, defense, and stress response. **(C)** *Cis*-elements were denoted by different colors according to their number. The darker the color, the higher the occurrence frequency, and the number indicates the number of *cis*-elements.

To clarify which hormonal, environmental stress, or developmental-related signal elements are involved in these *StSOS1s*, we performed a promoter analysis using the PlantCARE server. A large number of basic components were discovered in the upstream sequence (2000 bp) regions, including WRE3, GATA-motif, CAT-box and G-Box, but also P-box, TCA-element, AuxRR-core, TGACG-motif, ABRE and ERE hormonal response-related elements; as-1, LTR, ARE, GC-motif, MBS environmental stress-related components and A-box development-related elements (**Figures 2A, B**). Hormonal response elements were detected in the promoters of 37 potato *StSOS1* genes, including 15 SA, 19 MeJA, 26 ABA and 30 auxin response. The *cis*-elements involved in the GA response are present in all promoters of *StSOS1s*. The promoters of 10, 16, and 20 *StSOS1* genes contained MYB binding sites involved in low-temperature response, defense and stress response *cis*-elements and drought-inducibility, respectively (**Figure 2C**). These results suggest that the *StSOS1* genes may play a critical role not only in phytohormones, but also in biological and abiotic responses in the potato.

### 3.3 Gene structure and conserved motifs of StSOS1s

**Figure 3:**
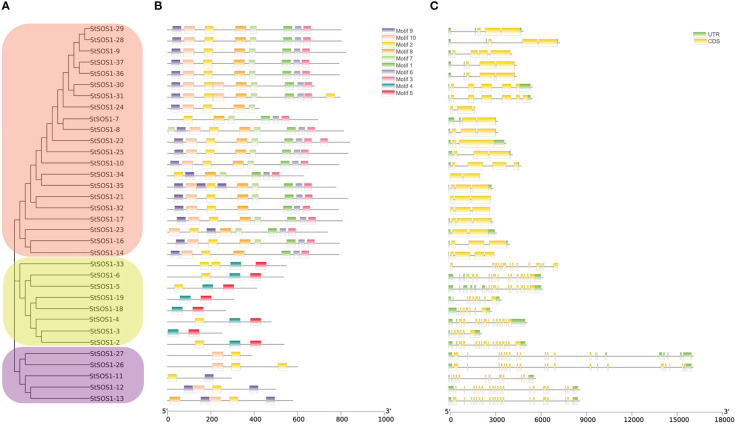
Phylogenetic relationships, structures, and motifs of members of the *StSOS1s* family [StSOS1-1 (1153 aa), StSOS1-15 (209 aa), and StSOS1-20 (252 aa) excepted]. **(A)** The phylogenetic tree of the StSOS1 proteins was constructed using the Maximum Likelihood method, which was based on conserved motifs and CDS/UTR structure. Different subgroups were represented by different background colors. **(B)** The conserved motifs of the StSOS1 proteins. Different patterns were represented by boxes of various colors, 5 ‘and 3’ represent the N and C ends. **(C)** Gene structures, exons and untranslated regions (UTR) are shown in green and yellow boxes, while black lines indicated introns. Phylogenetic trees, conserved motifs, and gene structures were predicted using TBtools, and their lengths were estimated using bottom ruler.

In order to better understand the relationship between the structure and function of these StSOS1 proteins, gene structure and conserved motifs were analyzed to construct individual phylogenies. Depending on the different branches of the evolutionary tree, it has been found that the motif architectures remain consistent within the same evolutionary branch, and thus they may have a similar function (**Figures 3A, B**). The results showed that the number of intron in *StSOS1* genes ranged from two (*StSOS1-22*, *StSOS1-23*, *StSOS1-34*) to 20 (*StSOS1-13*, *StSOS1-26*, *StSOS1-27*). Furthermore, closely related genes share a similar structural architectures with different introns lengths (**Figure 3C**). The shortest StSOS1 protein was just 209 aa in length (StSOS1-15), while the longest was StSOS1-1, with a length of 1153 aa (**Table 1**). The functional sites in the conserved motifs were analyzed using the Eukaryotic Linear Motif resource server (ELM) and the results showed that there was a great functional divergency among these sites and most of the functional sites are related to phosphorylation, kinase phosphorylation, binding and sorting signal responsible for the interaction (**Table S3**).

### 3.4 Expression characterization of *StSOS1s*

**Figure 4:**
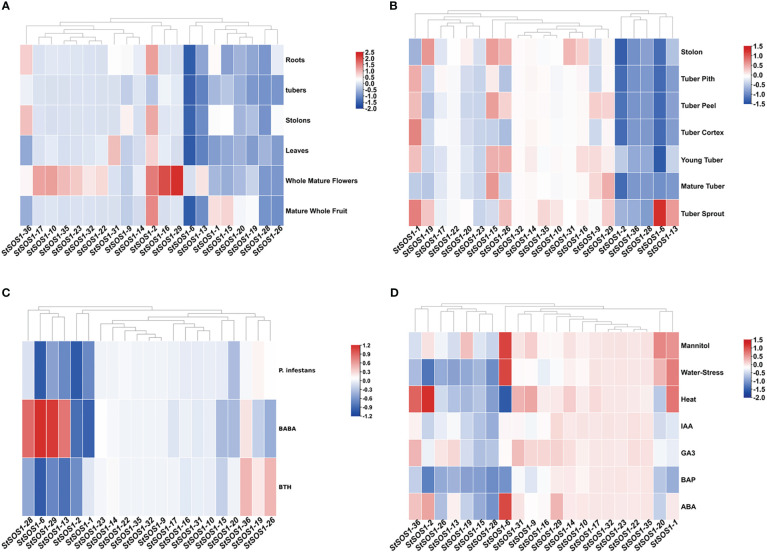
Expression levels of *StSOS1* genes in biotic, abiotic stress, and in different tissues and developmental stages. **(A)** Expression profiles of *StSOS1s* in different tissues and developmental stages. **(B)** Expression profiles of *StSOS1s* in different tissues and developmental stages of the potato tuber. **(C)** Expression of *StSOS1s* transcripts was altered in response to biotic stress. **(D)** Expression profiles of *StSOS1s* at abiotic stress and phytohormones. In the heat map, red, blue and white represent up-regulated, down-regulated, and unchanged (log_10_ ratio), respectively. Heat map and hierarchical clustering were performed by average linage (default) method.

To investigate the biological function of *StSOS1s* in different tissues, expression profiles of all identified *StSOS1* genes were analyzed in six different tissues, including roots, tubers, stolons, leaves, whole mature flowers, and mature whole fruit (**Figure 4A**). Of all the 21 *StSOS1* genes, *StSOS1-2* exhibits the highest levels of expression in almost all the tissues except the tubers. Some members of *StSOS1* exhibit highly tissue-specific expression, such as the expression of *StSOS1-10*, *StSOS1-16*, *StSOS1-17*, *StSOS1-19*, *StSOS1-22*, *StSOS1-23*, *StSOS1-32*, and *StSOS1-35* throughout the mature flower, suggesting that the *StSOS1* genes exhibit differential tissue-specific expression patterns. Then we analyzed spatio-temporal expression patterns in stolon, tuber pith, tuber peel, tuber cortex, young tuber, mature tuber and tuber sprout using RNA-seq data (**Figure 4B**). It showed that two genes (*StSOS1-14* and *StSOS1-32*) had a very low abundance in these tissues or organs. *StSOS1-16*, *StSOS1-19*, and *StSOS1-31* were predominantly expressed in stolon; *StSOS1-6* and *StSOS1-13* were predominantly expressed in tuber sprout. *StSOS1-1* was highly expressed in tuber pith, tuber peel, tuber cortex, young tuber and tuber sprouts. To have a better understand the function of *StSOS1s* under biotic stress, the expression pattern was observed responding to *Phytophthora infestans*, β-aminobutyric acid (BABA) and benzothiadiazole (BTH) treatment (**Figure 4C**). *StSOS1-2* was the only member to exhibit down-regulation under all three biotic stress conditions. Some genes show up-regulation, in particular one type of stress treatment; *StSOS1-6*, *StSOS1-13*, *StSOS1-28* and *StSOS1-29* showed up-regulation only in response to BABA treatment. For the abiotic stresses and phytohormones responsiveness of *StSOS1s*, we analyzed their transcript profiling in response to three abiotic stress and four phytohormone conditions mannitol, water-stress, heat, IAA, GA_3_, BAP and ABA (**Figure 4D**). *StSOS1-6* was found to be highly up-regulated in the three stress conditions of mannitol, water-stress and ABA. *StSOS1-10*, *StSOS1-17*, *StSOS1-22*, *StSOS1-23*, *StSOS1-32*, and *StSOS1-35* showed low or no expression in the eight tissues.

### 3.5 Gene ontology analyses of *StSOS1s*

**Figure 5:**
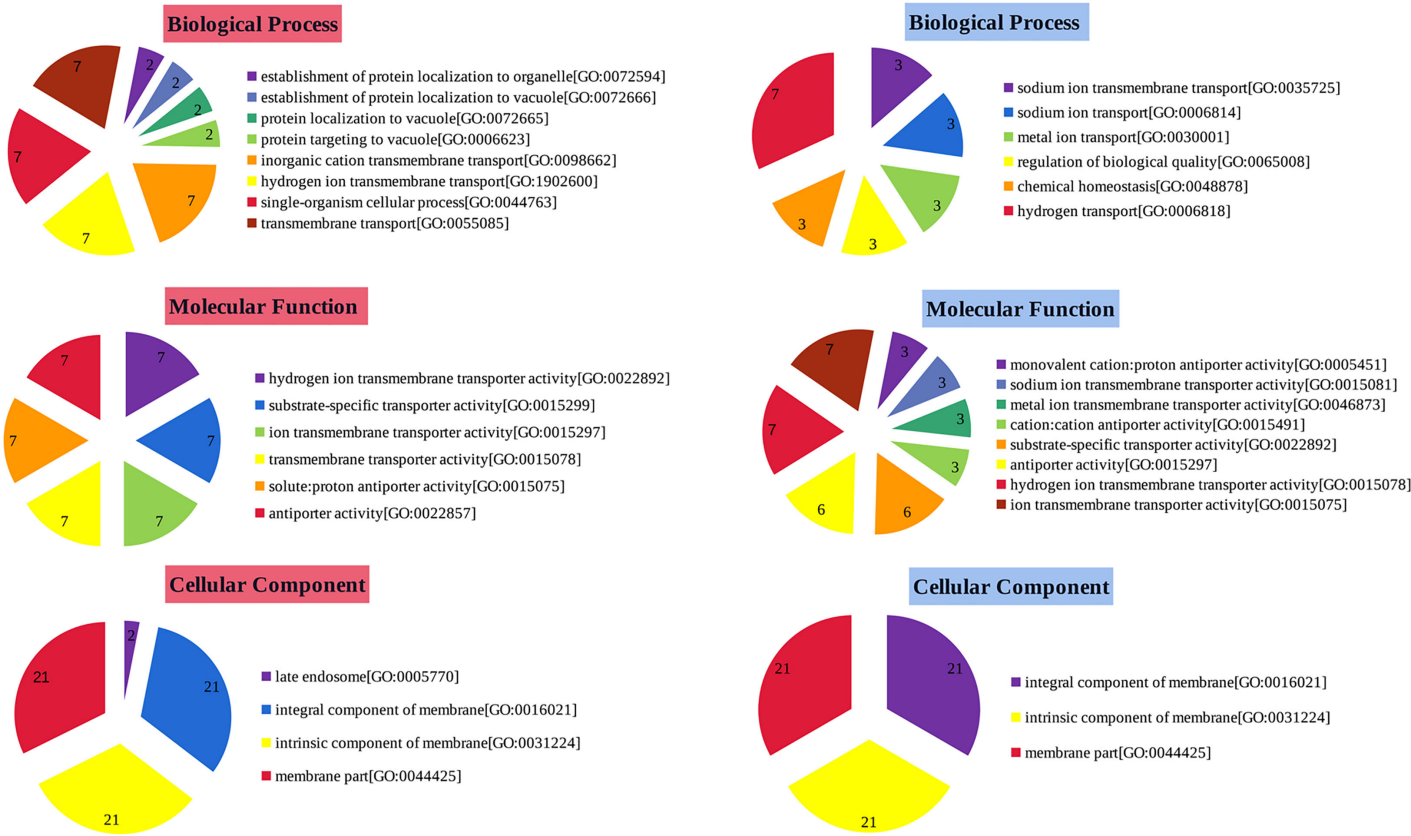
Gene ontology analyse of *StSOS1s*. Functional classification of up- and down-regulated genes by GO analysis into categories of Biological Process, Molecular Function and Cellular Component. The number in each pie represents the number of times the function (BP, CC, MF) was expressed in the data (abundance). GO-based classifications of up-regulated genes were shown in red, while those of down-regulated genes were shown in blue.

To identify functions of up and down-regulated genes, GO analysis was performed and genes belonging to different categories of Biological Processes (BP), Molecular Functions (MF) and Cellular Compartments (CC) were identified (**Figure 5**). The BP categorized results showed that the up-regulated genes were significantly enriched in transport and cellular process. For MF, these up-regulated states enriched in transport activity. Moreover, up-regulated genes in the CC category are significantly enriched in both membrane and membrane-like components. In addition, the most significantly enriched GO terms for down-regulated genes were detection of hydrogen transport (BP), and transporter activity and antiporter activity (MF). It is important to note that the membrane integral, the membrane intrinsic and membrane fraction are all present in both up- and down-regulated genes in CC. The difference is that the up-regulated genes have a late endosome while the down-regulated genes do not. In summary, most GO terms are involved in membrane transport and composition, suggesting that they are likely to play an important role in maintaining proper ion homeostasis in the cytoplasm.

The verification of PPI is a defining aspect of molecular biology. PPI analysis was conducted to analyze the interactions among the SOS1s (**Figure S2**). The biological pathways and cellular compartments (retrieved from the GO) associated with these proteins were similar. Here, the interaction network between 96 SOS1-related genes was also mapped using the STRING database and Cytoscape software for function analyse, seven clusters were identified, including the pathways of biological regulation, membrane, ion transmembrane transporter activity, calcium ion binding, potassium ion transmembrane transport, response to salt stress and cellular process (**Figure S3**). Only 25 *StSOS1s* interact with other genes, and the most PPI was observed between proteins involved in potassium ion transmembrane transport, response to salt stress and cellular processes. These studies inform the biochemical mechanism of *StSOS1* and provide a new reference for the interplay between ion homeostasis and transmembrane transport during plant salt tolerance.

### 3.6 Phylogenetic and collinearity analyses of *StSOS1s*

**Figure 6:**
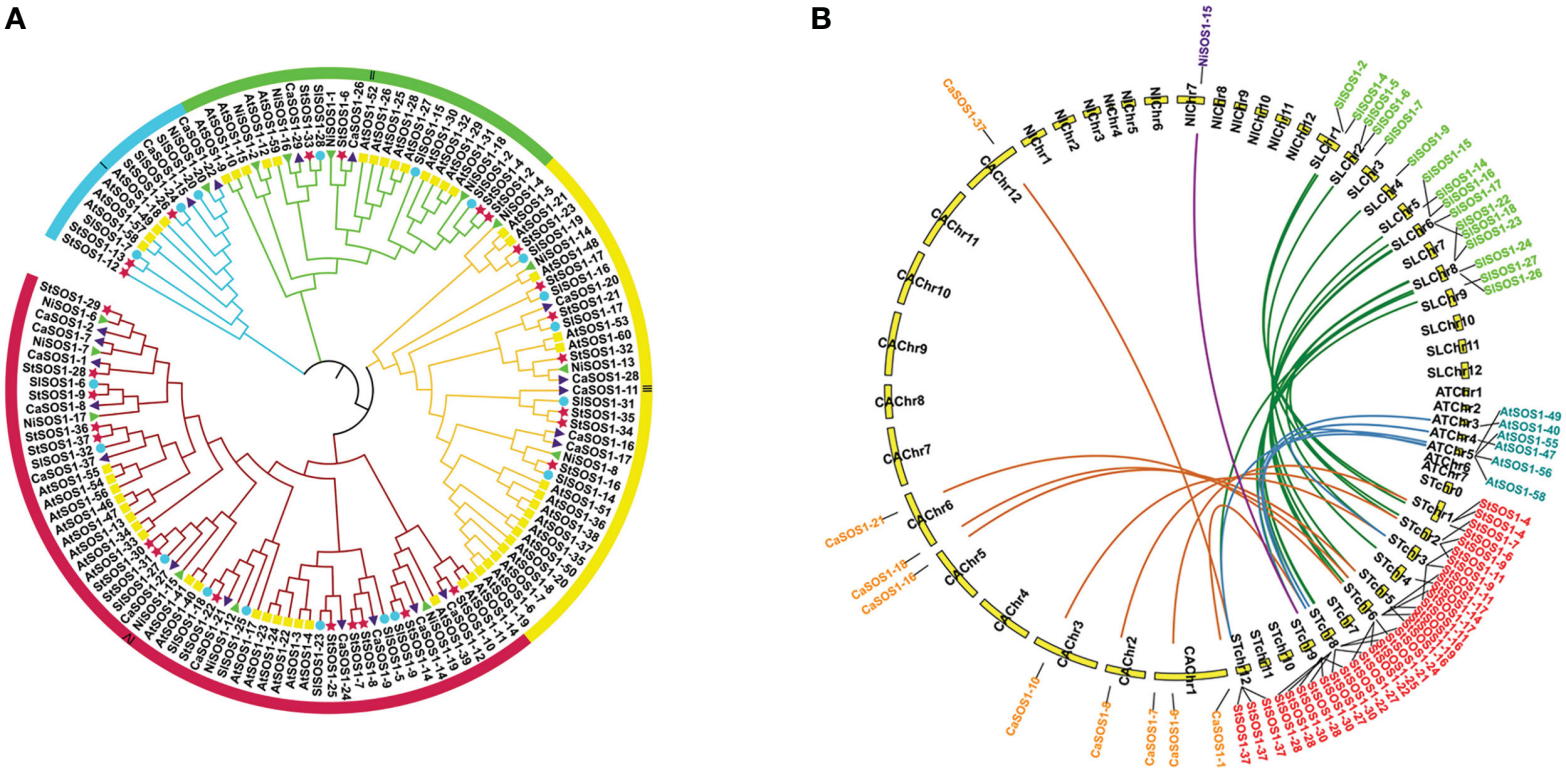
A Phylogenetic analysis of SOS1 proteins. **(A)** phylogenetic tree of SOS1 proteins was constructed with neighbor-junction (NJ) phylogenetic tree. The four subgroups were shown in different colors. The red stars represent potato SOS1s (StSOS1s), the green triangles represent tobacco SOS1s (NiSOSs), the purple triangles represent pepper SOS1s (CaSOSs), the yellow boxes represent Arabidopsis SOS1s (AtSOSs) and the blue circles represent tomato SOS1s (SlSOSs). **(B)** Collinearity analysis of SOS1s in potato and other plants. The green, orange, blue and purple lines in the background correspond to collinear gene pairs in potato and tomato, potato and pepper, and potato and Arabidopsis, potato and tobacco, respectively.

For the evolutionary relationship of SOS1s among Arabidopsis, tomato, pepper, potato and tobacco, we extracted and compared the protein sequences of SOS1s in these species, and constructed the phylogenetic tree of neighbor junction (NJ) (**Figure 6A**). Potato SOS1s are named based on their position relative to orthologs from four other species on the tree. 134 SOS1 candidates of five species were grouped into four distinct classes (I-IV) based on sequence conservation. Among them, the subgroup I had 13 members (11.19%), subgroup II 27 (20.14%) and subgroup III 37 (27.61%), respectively. The subgroup IV contained 57 genes and had the most members (42.54%). The phylogenetic relationships indicate that the SOS1 proteins in the potato are more strongly homologous to pepper and tomato than to Arabidopsis and tobacco. Gene duplication has always played a key role in the expansion of genes and the occurrence of novel functions of genes. To explore the evolution of *SOS1* genes, we studied the replication patterns of the five species and performed genetic correlation analysis (**Figure 6B**). The results showed that there were 17, 8, 5, and 1 *SOS1* members participating in the potato-tomato, potato-pepper, potato-Arabidopsis and potato-tobacco synteny relations, respectively. Among the above collinear gene pairs, *StSOS1-11* with *SlSOS1-23*, *CaSOS1-10*, *AtSOS1-58*, respectively; *StSOS1-28* with *SlSOS1-26*, *CaSOS1-1*, *AtSOS1-47*, respectively; and *StSOS1-37* with *SlSOS1-26*, *CaSOS1-37*, *AtSOS1-55*, respectively, had simultaneously collinear relations.

### 3.7 Expression analysis of *StSOS1* genes under different abiotic stresses

**Figure 7:**
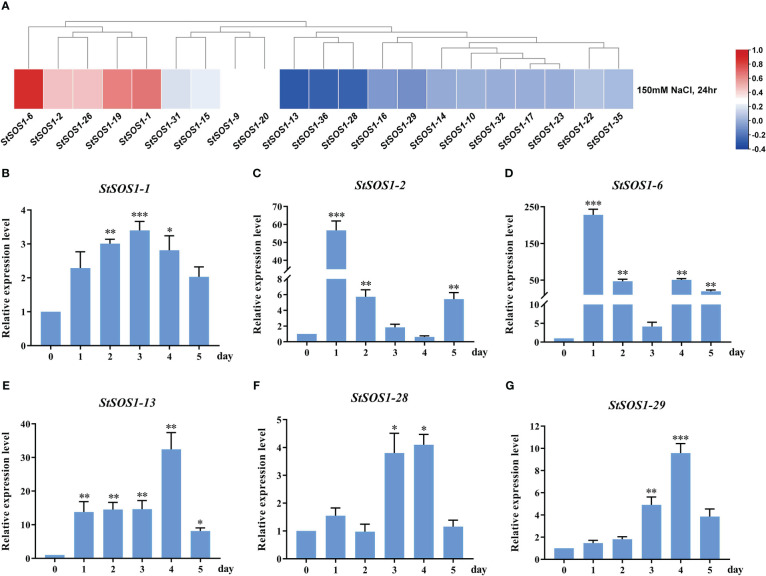
The expression pattern of *StSOS1s* under salt stress. **(A)** Expression profiles of *StSOS1s* at NaCl stress based on RNA seq-date. **(B-G)** RT-qPCR profiles of *StSOS1* genes under salt stress. The expression level of *StSOS1s* on control was normalized as “1”. The vertical bars indicate the standard error of the mean. Asterisks indicate significant differences based T test (*, *p* < 0.05, **, *p* < 0.01, ***, *p* < 0.001, ****, *p* < 0.0001).

The SOS pathway plays an important role in maintaining proper ion homeostasis in the cytoplasm and in regulating plant tolerance to salinity. However, there is limited information on *SOS1*’s response to potato salt stress. In order to investigate the potato response to salt stress, the *StSOS1* genes were analyzed using the transcriptomic data of potato exposed to NaCl treatment. Only 21 *StSOS1* genes showed differential gene expression pattern and were identified and visualized in a heat map (**Figure 7A**). Furthermore, six *StSOS1* genes in potato leaves of different grow stages under salt stress were randomly selected and quantitative analyzed by RT-qPCR (**Figures 7B–G**). These results suggested that these six genes were significantly differentially up-regulated under salt stress, which may positively regulate salt tolerance in the potato, this is not consistent with the heat map, which may be related with different levels of expression under different levels of salt stress treatment. *StSOS1-2*, *StSOS1-6* and *StSOS1-28* occurred two up-regulated expressions phenomenon under salt stress, this could be related to the response period of the SOS1 signaling pathway. Notably, the expression of *StSOS1-13* was 14-fold higher at 3 d after salt treatment compared to expression levels before salt stress, and then reached 32-fold higher at 4 d, suggesting that *StSOS1-13* may be an important candidate gene involved in the salt stress response.

**Figure 8:**
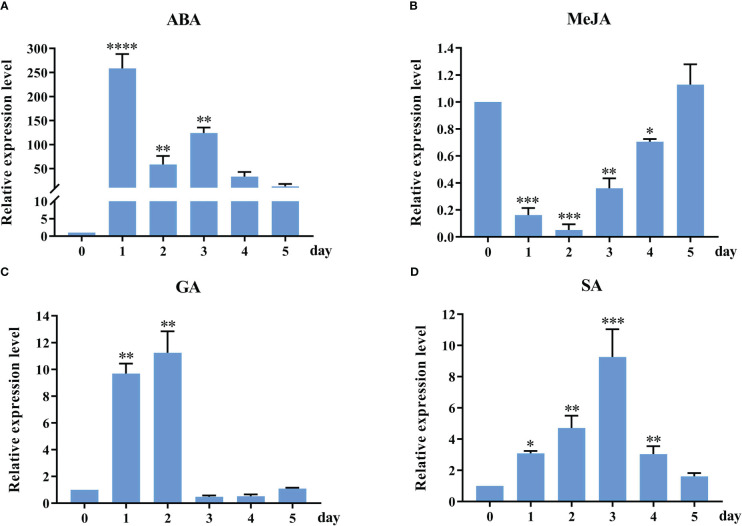
RT-qPCR profiles of *StSOS1-13* gene under phytohormone treatment. The *StSOS1-13* expression level of control was normalized as “1”. The vertical bars indicate the standard error of the mean. Asterisks indicate significant differences based on T test (*, *p* < 0.05, **, *p* < 0.01, ***, *p* < 0.001, ****, *p* < 0.0001).

To further understand potential function changes in *StSOS1-13* gene in response to abiotic stress, RT-qPCR was used to analyze the expression patterns of the selected *StSOS1-13* gene in phytohormone treatment (**Figure 8**). It was observed that the *StSOS1-13* was up-regulated on exposure to ABA, GA, and SA treatment, and the magnitude of up-regulation was higher in ABA treatment as compared to GA, SA treatment. Conversely, for the MeJA treatment, expression in the leaves decreased after 0-2 d and then increased continuously, with the highest levels of expression in the leaves at 5 d. Overall, these results indicated that *StSOS1-13* may play a critical regulatory role in response to abiotic stress.

### 3.8 Subcellular localization of StSOS1-13

**Figure 9:**
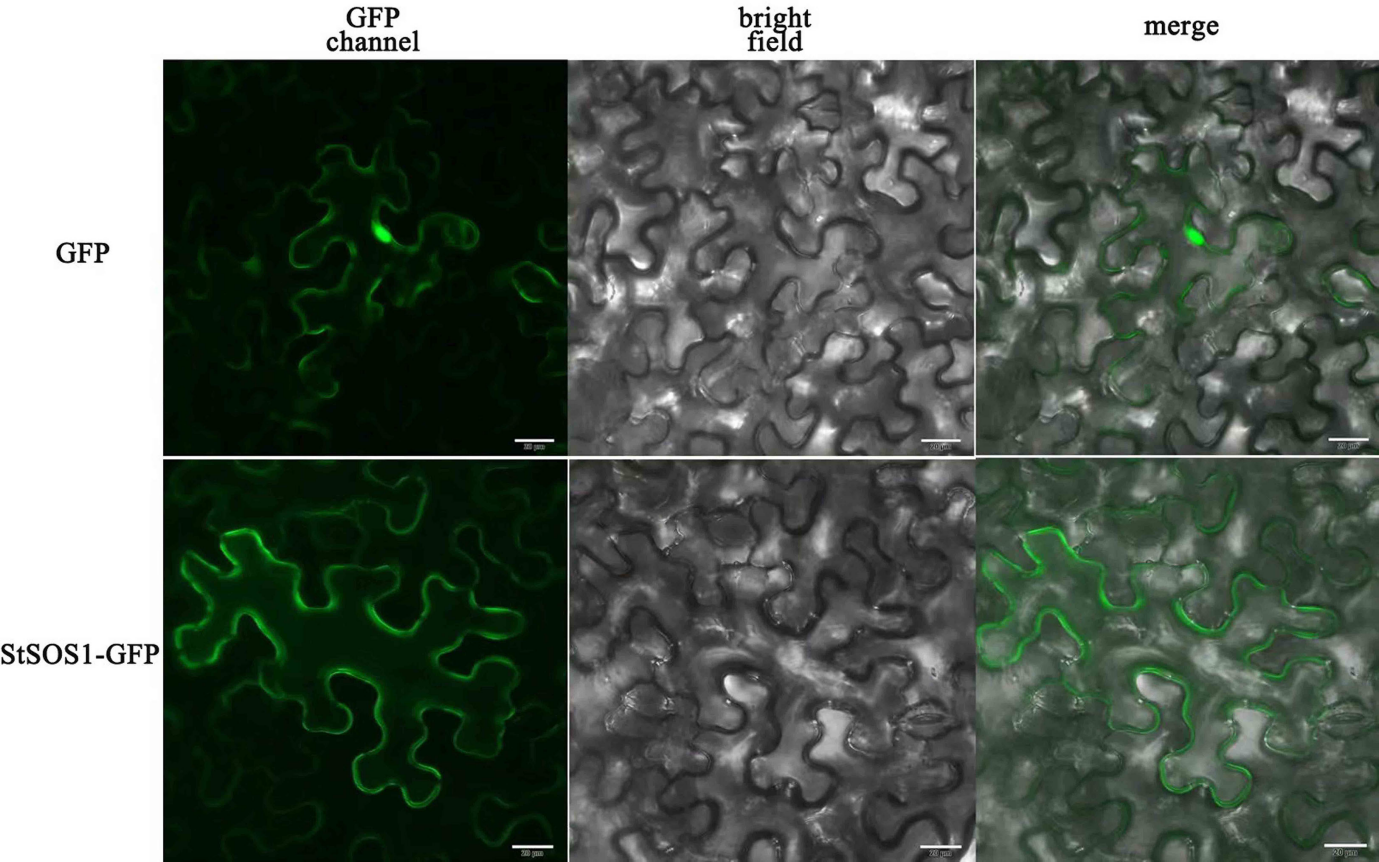
Subcellular localization of StSOS1-13 in *Nicotiana benthamiana*. The leaves were injected with a strain of *Agrobacterium tumefaciens* containing 35S::StSOS1-13-GFP, and the empty vector 35S::GFP were used as a control. After 48 h of injection, pStSOS1-13-GFP fusion protein and GFP alone transiently expressed separately in leaves, the dark field was green fluorescence and the white field was cell morphology, with Confocal combined detection. GFP, GFP fluorescence (green). Bright, bright fields. Merge, superimpose GFP and bright-field images. The experiment repeated three times with similar results. Scale bar, 20 µm.

Detecting the subcellular localization of StSOS1-13 is essential to elucidate their function. The subcellular localization of StSOS1-13 predicted by the Cell-PLoc 2.0 tool revealed that the StSOS1-13 protein was localized in the plasma membrane. To further verify the location of StSOS1-13 protein, the full-length coding sequence of StSOS1-13 deleted stop codon was fused with green fluorescence protein (GFP) and the transient expression was performed under the control of 35S promoter in tobacco. The results showed that the StSOS1-13 protein is localized in the plasma membrane (**Figure 9**), this is consistent with the result of bioinformatics analysis.

## 4 Discussion

Soil salinity is one of the most significant abiotic stresses faced by crop plants in agricultural fields worldwide (Świeżawska et al., 2018), reducing crop yield and production (Rolly et al., 2020). Plants have evolved the SOS pathway to achieve salt tolerance (Cha et al., 2022), the SOS pathway comprising *SOS1*, *SOS2* and *SOS3* has been proposed to regulate cellular signaling during salt stress to mediate ion homeostasis (Luo et al., 2022). *SOS1* is a critical salt tolerance determinant in plants (Świeżawska et al., 2018). *SOS1* genes have been reported to improve the tolerance to salt stresses in plants such as Arabidopsis (Wu et al., 1996), soybean (Zhang et al., 2022), and maize (Zhou et al., 2022). Potato is one of the most crucial crops in the world due to its nutritional quality (Takeuchi et al., 2022). The crop can also be used as a commercial health food because it is high in antioxidants, minerals, and dietary fibers (Kumar et al., 2021). In addition, potato plants are often subjected to various types of abiotic stress during growth and development (Yang et al., 2020; Kumar et al., 2021). It was reported that soil salinization negatively affected the growth and yield of potato crops, especially in arid and semi-arid climates (Li et al., 2022), which caused osmotic and oxidative stress, ion imbalance, mineral deficiency, and ion toxicity problems (Hamooh et al., 2021). Therefore, the selection and breeding of salt-tolerant genes has become a promising approach for improving the yield and adaptability of potato (Zhu et al., 2022). Previous studies have shown that a gene encoding SOS2 (PGSC0003DMG400006384) is up-regulated, indicating that this gene plays an active regulatory role in salt stress response. However, the complete SOS pathway for salt stress response in potato has not been established, and only a few genes of this pathway have been reported (Li Q. et al., 2020). The aim of this study is to screen for key *StSOS1* genes that are more sensitive to abiotic stress and to lay the groundwork for further unraveling the regulatory mechanisms of *SOS1* genes in potato.

In this study, a total of 37 *SOS1* family members were identified in potato (**Table 1**) and they locate in 10 of 12 chromosomes (**Figure 1**) which were significantly lower than Arabidopsis (60 *SOS1s* in **Figure 6A**). Gene duplication may explain the difference in the number of *SOS1* family members between the potato and Arabidopsis. A possible explanation for this is that *SOS1* genes in the potato may have a higher rate of gene loss than in Arabidopsis, and frequent gene loss has been reported in various plant species during genome duplication events (Li et al., 2020), indicating a key role of gene duplication over the course of evolution in various species (Zhang et al., 2021). Some of the duplicated genes may be retained in its descendants, which could provide the original genetic resource for the adaptive evolution of plants (Flagel and Wendel, 2009). The number of *SOS1* genes in the potato was similar to that in the pepper. Phylogenetic analysis demonstrated that the *Solanaceae SOS1* genes were generally classified into four clades (**Figure 6A**). Interestingly, four subfamilies were present in all five plant species, suggesting that genetic expansion occurred prior to the divergence of these plant species. By comparing the syntenic analysis of *SOS1* genes in potato and four other plants (tomato, pepper, Arabidopsis and tobacco), we found that the sequence similarity between the *SOS1* gene pairs within potato was much higher than that between the tomato and the pepper (**Figure 6B**), which is consistent with the phenomenon in chrysanthemum (Gao et al., 2016), indicating the similarity of evolutionary relationship among different species in the same group. The conserved motif analysis of *SOS1s* family revealed the occurrence of 10 conserved motifs (**Figure 3**) might be related to specific functions shared among *SOS1* family members. In addition, *StSOS1s* within the same subfamily share a high degree of similarity in exon-intron structures and conserved motifs. The loss and gain of introns may reflect evolutionary trends in genes with similar functions (Rogozin et al., 2003), which had been demonstrated in *Brassica juncea* (Cheng et al., 2019).

In salt-acclimated tobacco, the compartmentalization of Na^+^ in vacuoles may be mediated by vesicle transport (Garcia de la Garma et al., 2015), which represents an over-sensitive mechanism of the Na^+^/H^+^ antitransporter SOS1 to accommodate salt stress (Hamaji et al., 2009; Zhao S. et al., 2021). When the SOS signaling pathway is activated, the Na^+^/H^+^ antiport activity of *SOS1* is enhanced and the accumulated Na^+^ is transported out of the cell (Xie et al., 2022). For further functional analysis, we use GO annotation enrichment analysis to functionally annotate different *StSOS1s*. Gene ontology is a fundamental analysis that predicts the contribution of putative functions across living organisms. In the present study, GO analysis revealed the significant role of *StSOS1s* with cellular process, transport and component of membrane (**Figure 5**). To support this argument, we have constructed an additional PPI network with StSOS1 proteins as the core (**Figure S2**). Among the numerous functional modulated by the SOS1 network, there are the regulation pathways of biological regulation, membrane, ion transmembrane transporter activity, calcium ion binding, potassium ion transmembrane transport, response to salt stress and cellular process (**Figure S3**). Most of the *StSOS1* genes are involved in cellular transport process (**Figure S2**), suggesting that they probably play a vital role in maintaining appropriate ion homeostasis in the cytoplasm.

The *cis*-elements and functional characteristics of *SOS* genes promoters have been identified in many species, such as *Brassica juncea* var. *Tumida* (Cheng et al., 2019), *B. juncea* (Kaur et al., 2015), and Arabidopsis (Feki et al., 2015). To further explore the possible function of *SOS1s* in potato, we performed an analysis of *cis*-acting regulatory elements in the promoter region in this study. *Cis*-regulatory elements were found to include phytohormone (SA, MeJA, ABA, auxin, GA) and abiotic stresses (cold, defense and stress response, drought) (**Figure 2**), which is consistent with the report about the previous studies in other species. More importantly, the *cis*-elements involved in the GA response are present in the promoters of all *StSOS1s*, and more than half of the promoters of *StSOS1s* have MYB elements involved in drought-inducibility. Interestingly, in the heat map (**Figure 4D**), *StSOS1s* could be induced by both auxin and GA, two important plant hormones in regulation. Most of the *StSOS1s* notably up-regulate under both mannitol and NaCl stress conditions. Overall, the results presented above revealed that *StSOS1s* may play a significant role in the response to phytohormone and abiotic stresses.

In wheat, most *TaSOS1* genes expressed in different tissues, including shoots, leaves, spikes, and grains (Jiang et al., 2021). In Arabidopsis, *AtSOS1* promoter-driven GUS expressed primarily in the roots, inflorescences and leaves (Yang et al., 2009). Our results revealed *StSOS1-2* and *StSOS1-31* were specifically expressed in leaves whereas *StSOS1-10*, *StSOS1-16*, *StSOS1-17*, *StSOS1-19*, *StSOS1-22*, *StSOS1-23*, *StSOS1-32* and *StSOS1-35* had clear expression preference in whole mature flowers (**Figure 4A**). These suggested that the *StSOS1s* played a significant role in the growth and development of different potato organs. In addition, the *StSOS1s* had the similar tissue-specific expression patterns with the *AtSOS1s*, this suggested that the *SOS1* gene family played a conserved function in both Arabidopsis and potato.

Under salt stress, *SOS1* gene expression levels of *Populus euphratica* and *Chrysanthemum crassum* were up-regulated (Wu et al., 2007; Song et al., 2012). There were differences in *SOS1* gene expression in cotton at different time intervals (Akram et al., 2020). In this study, compared with the control, the expression level of *StSOS1s* in leaves was immediately up-regulated under salt stress, and the results of RT-qPCR of *StSOS1-1*, *StSOS1-2* and *StSOS1-6* were highly consistent with the results of heat map (**Figures 7B–D**). In addition, in wheat, the expression of *SOS1* in leaves under salt stress was consistent with mRNA abundance (Xu et al., 2008). However, the RT-qPCR results of *StSOS1-13*, *StSOS1-28* and *StSOS1-29* were contrary to the down-regulated results of heat map within 24 h (**Figures 7E–G**). In purslane (*Sesuvium portulacastrum*), the RT-qPCR results also differ from the heat map results. That is, the quantitative expression level of *SpSOS1* in roots increased sharply within 3-6 h and then decreased to the basic level, while the transcription abundance of *SOS1* in leaves did not change significantly within 48 h of NaCl treatment (Zhou et al., 2015). In addition, the expressions of *StSOS1-2*, *StSOS1-6* and *StSOS1-28* in leaves were up-regulated twice (**Figures 7C–D, F**). Similarly, the expression level of *GhSOS1* under salt stress also showed this phenomenon (Chen et al., 2017). In conclusion, the mechanism of *SOS1* in potato salt stress resistance is relatively complex and more studies are needed to determine the function of SOS1s in potato in the future.

Exogenous ABA, MeJA, SA treatment can improve the yield of potato (Pérez-Alonso et al., 2021). Under ABA stress, the expression of *BjSOS* genes increased with increasing stress duration in both contrasting genotypes (Nutan et al., 2018). Several reports have suggested co-expression of many stress-responsive genes at both salinity and ABA (Takahashi et al., 2004). Our results of RT-qPCR analysis indicated that the *StSOS1-13* was expressed under four phytohormone treatment (**Figure 8**). *StSOS1-13* was significantly up-regulated about 3, 9, and 250 times at 1 d in leaves under SA, GA, and ABA treatment, respectively, while *StSOS1-13*, was down-regulated under MeJA treatment. The promoter biological function is further corroborated by the expression analysis of *StSOS1-13* in response to hormonal stress. *StSOS1s* may regulate the expression of genes involved in the transduction of hormone signals, and thus participate in plant growth and development.

Studies have reported that the excessive Na^+^ ions in soil can cause imbalance *in vivo*, moisture deficiency and ion toxicity (Tester and Davenport, 2003), so some plants formed a Na^+^ efflux and Na^+^ segment processing. As a result, some plants have developed Na^+^ efflux and Na^+^ segment treatments to maintain low intracellular Na^+^ concentrations to accommodate the effects of salt stress on plant growth and development. The SOS pathway studied previously is a more classical salt signaling pathway (Chinnusamy et al., 2004). Arabidopsis salt-tolerant site SOS1 encodes Na^+^/H^+^ antiporter. Confocal imaging of a green fluorescent protein fusion protein of SOS1 in a transgenic Arabidopsis plant revealed that SOS1 is localized in the plasma membrane (Shi et al., 2002). SOS3 and SOS2, which are located in the cytoplasm, regulate SOS1 on the cytoplasmic membrane, which will therefore achieve an intracellular balance of Na^+^ (Hill et al., 2013). Protein subcellular location is key in determining the function and accumulation patterns of plant proteins (Hooper et al., 2020). In *Chrysanthemum crassum*, CcSOS1 was expressed close to the plasma membrane in transiently transformed onion epidermal cells (Song et al., 2012). Like the *A. thaliana* homologue *AtSOS1* (Shi et al., 2002), CcSOS1 is regulated by salinity, especially in the roots after stress, and could play an important role in salt tolerance in *C. crassum*. In rice (Gupta et al., 2021) and cotton (Guo et al., 2020), SOS1 genes were also predicted to express in plasma membrane. To investigate the subcellular localization of *StSOS1-13*, the cassette encoding *StSOS1-13*-Green Fluorescent protein (GFP) fusion protein driven by the CaMV 35S promoter (35S::*StSOS1-13*-GFP) was transformed into *Nicotiana benthamiana* leaves, and the fluorescence was observed using the confocal microscope. Fluorescence localization verified that the selected StSOS1-13 was expressed in the plasma membrane (**Figure 9**), demonstrating the reliability and accuracy of the predicted results.

## 5 Conclusions

This study provides a genome-wide analysis of the *StSOS1* genes, with 37 *StSOS1*s in the potato identified and divided into three subfamilies. We found that segmental and tandem duplication contribute to the expansion of *StSOS1* gene family. These *StSOS1s* phylogenetically cluster with *SlSOS1s* and *CaSOS1s.* The exon-intron structures and motifs of *StSOS1s* further suggest that the potato SOS1 proteins were highly conserved within the subfamilies. In addition, subcellular localization in *Nicotiana benthamiana* suggested that StSOS1-13 was located on the plasma membrane. The RT-qPCR results suggested the crucial role of the *StSOS1s* in response to salt and homologous stress, and suggested that some specific up-regulated genes such as *StSOS1-1*, *StSOS1-13*, and *StSOS1-29* would be potential candidates for potato salt-tolerant seeding. The results presented in this study will provide essential clues in elucidating the role of the *StSOS1s* in abiotic stress and the mechanisms underlying the tolerance to salt stress in potato mediated by the StSOS1 proteins.

## Data availability statement

The raw data supporting the conclusions of this article will be made available by the corresponding author GG (ggsxnu@126.com), without undue reservation. The potato RNA-Seq data in this article can be download in NCBI with accession number ERP000527.

## Author contributions

LL conceived and designed the study. LG analyzed and mapped the bioinformatics content, designed and performed the experimental work, interpreted and analyzed the data, and wrote the manuscript. YZ and ZH carried out the experimental work. WW, XZ, YW, XL, and SG helped to supplement the bioinformatics content and beautify the images. GG and WL supervised the project and critically revised the manuscript. All authors contributed to the article and approved the submitted version.

## Footnotes

1
http://plants.ensembl.org/index.html

2
http://pfam.xfam.org

3
http://hmmer.janelia.org/

4
http://www.ncbi.nlm.nih.gov/cdd

5
http://smart.emblheidelberg.de/

6
http://www.expasy.org/

7
https://services.healthtech.dtu.dk/services/NetPhos-3.1/

8
https://www.uniprot.org/

9
http://www.csbio.sjtu.edu.cn/bioinf/Cell-PLoc-2/

10
http://bioinformatics.psb.ugent.be/webtools/plantcare/html/

11
https://wordart.com

12
http://memesuite.org/

13
http://gsds.cbi.pku.edu.cn/

14
http://plantregmap.gao-lab.org/go.php

15
https://cn.string-db.org/

16
https://www.ncbi.nlm.nih.gov/tools/primer-blast/

## References

[B1] Świeżawska B. Duszyn M. Jaworski K. Szmidt-Jaworska A. (2018). Downstream targets of cyclic nucleotides in plants. Front. Plant Sci. 9. doi: 10.3389/fpls.2018.01428 PMC6174285 30327660

[B2] Akram U. Song Y. Liang C. Abid M. A. Askari M. Myat A. A.. (2020). Genome-wide characterization and expression analysis of NHX gene family under salinity stress in *Gossypium barbadense* and its comparison with *Gossypium hirsutum* . Genes (Basel) 11, 803. doi: 10.3390/genes11070803 32708576 PMC7397021

[B3] Ali A. Alexandersson E. Sandin M. Resjö S. Lenman M. Hedley P.. (2014). Quantitative proteomics and transcriptomics of potato in response to *Phytophthora infestans* in compatible and incompatible interactions. BMC Genomics 15, 497. doi: 10.1186/1471-2164-15-497 24947944 PMC4079953

[B4] Ali A. Raddatz N. Pardo J. M. Yun D. J. (2021). HKT sodium and potassium transporters in *Arabidopsis thaliana* and related halophyte species. Physiol. Plant 171, 546–558. doi: 10.1111/ppl.13166 32652584 PMC8048799

[B5] Brindha C. Vasantha S. Raja A. K. Tayade A. S. (2021). Characterization of the salt overly sensitive pathway genes in sugarcane under salinity stress. Physiol. Plant 171, 677–687. doi: 10.1111/ppl.13245 33063359

[B6] Ceci A. T. Franceschi P. Serni E. Perenzoni D. Oberhuber M. Robatscher P.. (2022). Metabolomic characterization of pigmented and non-pigmented potato cultivars using a joint and individual variation explained (JIVE). Foods 11, 1708. doi: 10.3390/foods11121708 35741905 PMC9223171

[B7] Cha J. Y. Kim J. Jeong S. Y. Shin G. I. Ji M. G. Hwang J. W.. (2022). The Na(+)/H(+) antiporter SALT OVERLY SENSITIVE 1 regulates salt compensation of circadian rhythms by stabilizing GIGANTEA in *Arabidopsis* . Proc. Natl. Acad. Sci. U. S. A. 119, e2207275119. doi: 10.1073/pnas.2207275119 35939685 PMC9388102

[B8] Chen C. Chen H. Zhang Y. Thomas H. R. Frank M. H. He Y.. (2020). TBtools: an integrative toolkit developed for interactive analyses of big biological data. Mol. Plant 13, 1194–1202. doi: 10.1016/j.molp 32585190

[B9] Chen X. Lu X. Shu N. Wang D. Wang S. Wang J.. (2017). *GhSOS1*, a plasma membrane Na^+^/H^+^ antiporter gene from upland cotton, enhances salt tolerance in transgenic *Arabidopsis thaliana* . PloS One 12, e0181450. doi: 10.1371/journal.pone.0181450 28723926 PMC5517032

[B10] Cheng C. Zhong Y. Wang Q. Cai Z. Wang D. Li C. (2019). Genome-wide identification and gene expression analysis of SOS family genes in tuber mustard (*Brassica juncea* var. *tumida*). PloS One 14, e0224672. doi: 10.1371/journal.pone 31710609 PMC6844470

[B11] Chinnusamy V. Schumaker K. Zhu J. K. (2004). Molecular genetic perspectives on cross-talk and specificity in abiotic stress signalling in plants. J. Exp. Bot. 55, 225–236. doi: 10.1093/jxb/erh005 14673035

[B12] Contreras-Moreira B. Naamati G. Rosello M. Allen J. E. Hunt S. E. Muffato M.. (2022). Scripting analyses of genomes in ensembl plants. Methods Mol. Biol. 2443, 27–55. doi: 10.1007/978-1-0716-2067-0_2 35037199 PMC7614177

[B13] Dahal K. Li X. Q. Tai H. Creelman A. Bizimungu B. (2019). Improving potato stress tolerance and tuber yield under a climate change scenario - a current overview. Front. Plant Sci. 10. doi: 10.3389/fpls.2019.00563 PMC6527881 31139199

[B14] Fayez A. G. Esmaiel N. N. Salem S. M. Ashaat E. A. El-Saiedi S. A. El Ruby M. O. (2022). miR-454-3p and miR-194-5p targeting cardiac sarcolemma ion exchange transcripts are potential noninvasive diagnostic biomarkers for childhood dilated cardiomyopathy in Egyptian patients. Egypt Heart J. 74, 65. doi: 10.1186/s43044-022-00300-x 36076093 PMC9458794

[B15] Feki K. Brini F. Ben Amar S. Saibi W. Masmoudi K. (2015). Comparative functional analysis of two wheat Na(+)/H (+) antiporter SOS1 promoters in *Arabidopsis thaliana* under various stress conditions. J. Appl. Genet. 56, 15–26. doi: 10.1007/s13353-014-0228-7 25081835

[B16] Flagel L. E. Wendel J. F. (2009). Gene duplication and evolutionary novelty in plants. New Phytol. 183, 557–564. doi: 10.1111/j.1469-8137 19555435

[B17] Gao J. Sun J. Cao P. Ren L. Liu C. Chen S.. (2016). Variation in tissue na(+) content and the activity of *SOS1* genes among two species and two related genera of chrysanthemum. BMC Plant Biol. 16, 98. doi: 10.1186/s12870-016-0781-9 27098270 PMC4839091

[B18] Garcia de la Garma J. Fernandez-Garcia N. Bardisi E. Pallol B. Asensio-Rubio J. S. Bru R.. (2015). New insights into plant salt acclimation: the roles of vesicle trafficking and reactive oxygen species signalling in mitochondria and the endomembrane system. New Phytol. 205, 216–239. doi: 10.1111/nph.12997 25187269

[B19] Goodstein D. M. Shu S. Howson R. Neupane R. Hayes R. D. Fazo J.. (2012). Phytozome: a comparative platform for green plant genomics. Nucleic Acids Res. 40 (Database issue), D1178–D1186. doi: 10.1093/nar/gkr944 22110026 PMC3245001

[B20] Guo W. Li G. Wang N. Yang C. Zhao Y. Peng H.. (2020). A Na(+)/H(+) antiporter, K2-NhaD, improves salt and drought tolerance in cotton (*Gossypium hirsutum* l.). Plant Mol. Biol. 102, 553–567. doi: 10.1007/s11103-020-00969-1 31989373

[B21] Gupta B. K. Sahoo K. K. Anwar K. Nongpiur R. C. Deshmukh R. Pareek A.. (2021). Silicon nutrition stimulates salt-overly sensitive (SOS) pathway to enhance salinity stress tolerance and yield in rice. Plant Physiol. Biochem. 166, 593–604. doi: 10.1016/j.plaphy.2021.06.010 34186283

[B22] Hamaji K. Nagira M. Yoshida K. Ohnishi M. Oda Y. Uemura T.. (2009). Dynamic aspects of ion accumulation by vesicle traffic under salt stress in arabidopsis. Plant Cell Physiol. 50, 2023–2033. doi: 10.1093/pcp/pcp143 19880402

[B23] Hamooh B. T. Sattar F. A. Wellman G. Mousa M. A. A. (2021). Metabolomic and biochemical analysis of two potato (*Solanum tuberosum* l.) cultivars exposed to *in vitro* osmotic and salt stresses. Plants (Basel) 10, 98. doi: 10.3390/plants10010098 33418964 PMC7825055

[B24] He F. Shi Y. J. Chen Q. Li J. L. Niu M. X. Feng C. H.. (2022). Genome-wide investigation of the *PtrCHLP* family reveals that *PtrCHLP3* actively mediates poplar growth and development by regulating photosynthesis. Front. Plant Sci. 13. doi: 10.3389/fpls PMC9127975 35620683

[B25] Hill C. B. Jha D. Bacic A. Tester M. Roessner U. (2013). Characterization of ion contents and metabolic responses to salt stress of different arabidopsis *AtHKT1;1* genotypes and their parental strains. Mol. Plant 6, 350–368. doi: 10.1093/mp/sss125 23132143

[B26] Hooper C. M. Castleden I. R. Aryamanesh N. Black K. Grasso S. V. Millar A. H. (2020). CropPAL for discovering divergence in protein subcellular location in crops to support strategies for molecular crop breeding. Plant J. 104, 812–827. doi: 10.1111/tpj.14961 32780488

[B27] Ilzhöfer D. Heinzinger M. Rost B. (2022). SETH predicts nuances of residue disorder from protein embeddings. Front. Bioinform. 2. doi: 10.3389/fbinf PMC9580958 36304335

[B28] Jiang W. Pan R. Buitrago S. Wu C. Abou-Elwafa S. F. Xu Y.. (2021). Conservation and divergence of the *TaSOS1* gene family in salt stress response in wheat (*Triticum aestivum* l.). Physiol. Mol. Biol. Plants. 27, 1245–1260. doi: 10.1007/s12298-021-01009-y 34177146 PMC8212347

[B29] Kaur C. Kumar G. Kaur S. Ansari M. W. Pareek A. Sopory S. K.. (2015). Molecular cloning and characterization of salt overly sensitive gene promoter from *Brassica juncea* (*BjSOS2*). Mol. Biol. Rep. 42, 1139–1148. doi: 10.1007/s11033-015-3851-4 25633281

[B30] Keisham M. Mukherjee S. Bhatla S. C. (2018). Mechanisms of sodium transport in plants-progresses and challenges. Int. J. Mol. Sci. 19, 647. doi: 10.3390/ijms19030647 29495332 PMC5877508

[B31] Koul A. Sharma D. Kaul S. Dhar M. K. (2019). Identification and in silico characterization of *cis*-acting elements of genes involved in carotenoid biosynthesis in tomato. 3 Biotech. 9, 287. doi: 10.1007/s13205-019-1798-1 PMC6595038 31297303

[B32] Kumar P. Kumar P. Sharma D. Verma S. K. Halterman D. Kumar A. (2021). Genome-wide identification and expression profiling of basic leucine zipper transcription factors following abiotic stresses in potato (*Solanum tuberosum* l.). PloS One 16, e0247864. doi: 10.1371/journal.pone.0247864 33711039 PMC7954325

[B33] Li H. Guan H. Zhuo Q. Wang Z. Li S. Si J.. (2020). Genome-wide characterization of the abscisic *acid-*, *stress-* and *ripening-induced* (*ASR*) gene family in wheat (*Triticum aestivum* l.). Biol. Res. 53, 23. doi: 10.1186/s40659-020-00291-6 32448297 PMC7247183

[B34] Li J. Li X. Han P. Liu H. Gong J. Zhou W.. (2021). Genome-wide investigation of *bHLH* genes and expression analysis under different biotic and abiotic stresses in *Helianthus annuus* l. Int. J. Biol. Macromol. 189, 72–83. doi: 10.1016/j.ijbiomac 34411617

[B35] Li Q. Qin Y. Hu X. Jin L. Li G. Gong Z.. (2022). Physiology and gene expression analysis of potato (*Solanum tuberosum* l.) in salt stress. Plants (Basel) 11, 1565. doi: 10.3390/plants11121565 35736717 PMC9229698

[B36] Li Q. Qin Y. Hu X. Li G. Ding H. Xiong X.. (2020). Transcriptome analysis uncovers the gene expression profile of salt-stressed potato (*Solanum tuberosum* l.). Sci. Rep. 10, 5411. doi: 10.1038/s41598-020-62057-0 32214109 PMC7096413

[B37] Liang Y. Wan N. Cheng Z. Mo Y. Liu B. Liu H.. (2017). Whole-genome identification and expression pattern of the vicinal oxygen chelate family in rapeseed (*Brassica napus* l.). Front. Plant Sci. 8. doi: 10.3389/fpls.2017.00745 PMC5422514 28536594

[B38] Luo B. Guang M. Yun W. Ding S. Ren S. Gao H. (2022). Camellia sinensis chloroplast fluoride efflux gene *CsABCB9* is involved in the fluoride tolerance mechanism. Int. J. Mol. Sci. 23, 7756. doi: 10.3390/ijms23147756 35887104 PMC9317437

[B39] Ma R. Liu W. Li S. Zhu X. Yang J. Zhang N.. (2021). Genome-wide identification, characterization and expression analysis of the *CIPK* gene family in potato (*Solanum tuberosum* l.) and the role of *StCIPK10* in response to drought and osmotic stress. Int. J. Mol. Sci. 22, 13535. doi: 10.3390/ijms222413535 34948331 PMC8708990

[B40] Mo F. Li L. Zhang C. Yang C. Chen G. Niu Y.. (2022). Genome-wide analysis and expression profiling of the *Phenylalanine ammonia-lyase* gene family in *Solanum tuberosum* . Int. J. Mol. Sci. 23, 6833. doi: 10.3390/ijms23126833 35743276 PMC9224352

[B41] Naureen U. Khosa A. N. Mukhtar M. A. Nabi F. Ahmed N. Saleem M. (2023). Genetic biodiversity and posttranslational modifications of protease serine endopeptidase in different strains of *Sordaria fimicola* . BioMed. Res. Int. 2023, 2088988. doi: 10.1155/2023/2088988 36814796 PMC9940969

[B42] Núñez-Ramírez R. Sánchez-Barrena M. J. Villalta I. Vega J. F. Pardo J. M. Quintero F. J.. (2012). Structural insights on the plant salt-overly-sensitive 1 (SOS1) Na(+)/H(+) antiporter. J. Mol. Biol. 424, 283–294. doi: 10.1016/j.jmb.2012.09.015 23022605

[B43] Nutan K. K. Kumar G. Singla-Pareek S. L. Pareek A. (2018). A salt overly sensitive pathway member from *Brassica juncea* BjSOS3 can functionally complement *ΔAtsos3* in arabidopsis. Curr. Genomics 19, 60–69. doi: 10.2174/1389202918666170228133621 29491733 PMC5817878

[B44] Pérez-Alonso M. M. Ortiz-García P. Moya-Cuevas J. Pollmann S. (2021). Mass spectrometric monitoring of plant hormone cross talk during biotic stress responses in potato (*Solanum tuberosum* l.). Methods Mol. Biol. 2354, 143–154. doi: 10.1007/978-1-0716-1609-3_7 34448159

[B45] Rogozin I. B. Wolf Y. I. Sorokin A. V. Mirkin B. G. Koonin E. V. (2003). Remarkable interkingdom conservation of intron positions and massive, lineage-specific intron loss and gain in eukaryotic evolution. Curr. Biol. 13, 1512–1517. doi: 10.1016/s0960-9822(03)00558-x 12956953

[B46] Rolly N. K. Imran Q. M. Lee I. J. Yun B. W. (2020). Salinity stress-mediated suppression of expression of salt overly sensitive signaling pathway genes suggests negative regulation by *AtbZIP62* transcription factor in *Arabidopsis thaliana* . Int. J. Mol. Sci. 21, 1726. doi: 10.3390/ijms21051726 32138325 PMC7084470

[B47] Sharma B. Saxena H. Negi H. (2021). Genome-wide analysis of HECT E3 ubiquitin ligase gene family in *Solanum lycopersicum* . Sci. Rep. 11, 15891. doi: 10.1038/s41598-021-95436-2 34354159 PMC8342558

[B48] Shi H. Ishitani M. Kim C. Zhu J. K. (2000). The *Arabidopsis thaliana* salt tolerance gene *SOS1* encodes a putative Na^+^/H^+^ antiporter. Proc. Natl. Acad. Sci. U. S. A. 97, 6896–6901. doi: 10.1073/pnas.120170197 10823923 PMC18772

[B49] Shi H. Lee B. H. Wu S. J. Zhu J. K. (2003). Overexpression of a plasma membrane Na^+^/H^+^ antiporter gene improves salt tolerance in *Arabidopsis thaliana* . Nat. Biotechnol. 21, 81–85. doi: 10.1038/nbt766 12469134

[B50] Shi H. Quintero F. J. Pardo J. M. Zhu J. K. (2002). The putative plasma membrane Na(+)/H(+) antiporter SOS1 controls long-distance na(+) transport in plants. Plant Cell. 14, 465–477. doi: 10.1105/tpc.010371 11884687 PMC152925

[B51] Song A. Lu J. Jiang J. Chen S. Guan Z. Fang W.. (2012). Isolation and characterisation of *Chrysanthemum crassum SOS1*, encoding a putative plasma membrane Na(+)/H(+) antiporter. Plant Biol. (Stuttg). 14, 706–713. doi: 10.1111/j.1438-8677 22404736

[B52] Sun H. Ren M. Zhang J. (2022). Genome-wide identification and expression analysis of fibrillin (*FBN*) gene family in tomato (*Solanum lycopersicum* l.). PeerJ 10, e13414. doi: 10.7717/peerj.13414 35573169 PMC9097668

[B53] Takahashi S. Seki M. Ishida J. Satou M. Sakurai T. Narusaka M.. (2004). Monitoring the expression profiles of genes induced by hyperosmotic, high salinity, and oxidative stress and abscisic acid treatment in arabidopsis cell culture using a full-length cDNA microarray. Plant Mol. Biol. 56, 29–55. doi: 10.1007/s11103-004-2200-0 15604727

[B54] Takeuchi A. Akatsu Y. Asahi T. Okubo Y. Ohnuma M. Teramura H.. (2022). Procedure for the efficient acquisition of progeny seeds from crossed potato plants grafted onto tomato. Plant Biotechnol. (Tokyo). 39, 195–197. doi: 10.5511/plantbiotechnology.21.1119a 35937528 PMC9300436

[B55] Tester M. Davenport R. (2003). Na^+^ tolerance and na^+^ transport in higher plants. Ann. Bot. 91, 503–527. doi: 10.1093/aob/mcg058 12646496 PMC4242248

[B56] Wang T. Gao X. Chen S. Li D. Chen S. Xie M.. (2021). Genome-wide identification and expression analysis of ethylene responsive factor family transcription factors in *Juglans regia* . PeerJ 9, e12429. doi: 10.7717/peerj.12429 34820183 PMC8607932

[B57] Wang Z. Hong Y. Li Y. Shi H. Yao J. Liu X.. (2021). Natural variations in *SlSOS1* contribute to the loss of salt tolerance during tomato domestication. Plant Biotechnol. J. 19, 20–22. doi: 10.1111/pbi.13443 32634852 PMC7769236

[B58] Wang J. Zhang Y. Xu N. Zhang H. Fan Y. Rui C.. (2021). Genome-wide identification of *CK* gene family suggests functional expression pattern against Cd(2+) stress in *Gossypium hirsutum* l. Int. J. Biol. Macromol. 188, 272–282. doi: 10.1016/j.ijbiomac 34364943

[B59] Wu Y. Ding N. Zhao X. Zhao M. Chang Z. Liu J.. (2007). Molecular characterization of *PeSOS1*: the putative Na(+)/H (+) antiporter of *Populus euphratica* . Plant Mol. Biol. 65, 1–11. doi: 10.1007/s11103-007-9170-y 17605111

[B60] Wu S. J. Ding L. Zhu J. K. (1996). SOS1, a genetic locus essential for salt tolerance and potassium acquisition. Plant Cell. 8, 617–627. doi: 10.1105/tpc.8.4.617 12239394 PMC161124

[B61] Xiang X. H. Wu X. R. Chao J. T. Yang M. L. Yang F. Chen G.. (2016). Genome-wide identification and expression analysis of the *WRKY* gene family in common tobacco (*Nicotiana tabacum* l.). Yi Chuan. 38, 840–856. doi: 10.16288/j.yczz 27644745

[B62] Xie Q. Zhou Y. Jiang X. (2022). Structure, function, and regulation of the plasma membrane Na(+)/H(+) antiporter salt overly sensitive 1 in plants. Front. Plant Sci. 13. doi: 10.3389/fpls PMC9009148 35432437

[B63] Xu H. Jiang X. Zhan K. Cheng X. Chen X. Pardo J. M.. (2008). Functional characterization of a wheat plasma membrane Na^+^/H^+^ antiporter in yeast. Arch. Biochem. Biophys. 473, 8–15. doi: 10.1016/j.abb 18316035

[B64] Yang Q. Chen Z. Z. Zhou X. F. Yin H. B. Li X. Xin X. F.. (2009). Overexpression of *SOS* (*Salt overly sensitive*) genes increases salt tolerance in transgenic *Arabidopsis* . Mol. Plant 2, 22–31. doi: 10.1093/mp/ssn058 19529826 PMC2639737

[B65] Yang G. Xu C. Varjani S. Zhou Y. Wc Wong J. Duan G. (2022). Metagenomic insights into improving mechanisms of Fe(0) nanoparticles on volatile fatty acids production from potato peel waste anaerobic fermentation. Bioresour. Technol. 361, 127703. doi: 10.1016/j.biortech.2022.127703 35907599

[B66] Yang X. Yuan J. Luo W. Qin M. Yang J. Wu W.. (2020). Genome-wide identification and expression analysis of the class III peroxidase gene family in potato (*Solanum tuberosum* l.). Front. Genet. 11. doi: 10.3389/fgene.2020.593577 PMC7744636 33343634

[B67] You X. Nasrullah, Wang D. Mei Y. Bi J. Liu S.. (2022). N(7) -SSPP fusion gene improves salt stress tolerance in transgenic arabidopsis and soybean through ROS scavenging. Plant Cell Environ. 45, 2794–2809. doi: 10.1111/pce.14392 35815549

[B68] Yu R. M. Suo Y. Y. Yang R. Chang Y. N. Tian T. Song Y. J.. (2021). *StMBF1c* positively regulates disease resistance to *Ralstonia solanacearum via* it’s primary and secondary upregulation combining expression of *StTPS5* and resistance marker genes in potato. Plant Sci. 307, 110877. doi: 10.1016/j.plantsci 33902863

[B69] Zhang M. Cao J. Zhang T. Xu T. Yang L. Li X.. (2022). A putative plasma membrane Na(+)/H(+) antiporter *GmSOS1* is critical for salt stress tolerance in *Glycine max* . Front. Plant Sci. 13. doi: 10.3389/fpls.2022.870695 PMC9149370 35651772

[B70] Zhang Q. Hou S. Sun Z. Chen J. Meng J. Liang D.. (2021). Genome-wide identification and analysis of the *MADS-box* gene family in *Theobroma cacao* . Genes (Basel) 12. doi: 10.3389/fpls.2022.870695 PMC8622960 34828404

[B71] Zhang C. Liu S. Liu D. Guo F. Yang Y. Dong T.. (2022). Genome-wide survey and expression analysis of GRAS transcription factor family in sweetpotato provides insights into their potential roles in stress response. BMC Plant Biol. 22, 232. doi: 10.1186/s12870-022-03618-5 35524176 PMC9074257

[B72] Zhao C. William D. Sandhu D. (2021). Isolation and characterization of salt overly sensitive family genes in spinach. Physiol. Plant 171, 520–532. doi: 10.1111/ppl.13125 32418228

[B73] Zhao S. Zhang Q. Liu M. Zhou H. Ma C. Wang X. X. X. P. (2021). Regulation of plant responses to salt stress. Int. J. Mol. Sci. 22, 4609. doi: 10.3390/ijms22094609 33924753 PMC8125386

[B74] Zhou X. Li J. Wang Y. Liang X. Zhang M. Lu M.. (2022). The classical SOS pathway confers natural variation of salt tolerance in maize. New Phytol. 236, 479–494. doi: 10.1111/nph.18278 35633114

[B75] Zhou Y. Yin X. Duan R. Hao G. Guo J. Jiang X. (2015). *SpAHA1* and *SpSOS1* coordinate in transgenic yeast to improve salt tolerance. PloS One 10, e0137447. doi: 10.1371/journal.pone.0137447 26340746 PMC4560418

[B76] Zhu L. Li M. Huo J. Lian Z. Liu Y. Lu L.. (2021). Overexpression of *NtSOS2* from halophyte plant *N. tangutorum* enhances tolerance to salt stress in arabidopsis. Front. Plant Sci. 12. doi: 10.3389/fpls.2021.716855 PMC8450600 34552607

[B77] Zhu J. K. Liu J. Xiong L. (1998). Genetic analysis of salt tolerance in arabidopsis. evidence for a critical role of potassium nutrition. Plant Cell. 10, 1181–1191. doi: 10.1105/tpc.10.7.1181 9668136 PMC144057

[B78] Zhu X. Wang F. Li S. Feng Y. Yang J. Zhang N.. (2022). Calcium-dependent protein kinase 28 maintains potato photosynthesis and its tolerance under water deficiency and osmotic stress. Int. J. Mol. Sci. 23, 8795. doi: 10.3390/ijms23158795 35955930 PMC9368905

