# Supplementary material for: Genome-wide characterization of SOS1 gene family in potato (Solanum tuberosum) and expression analyses under salt and hormone stress
Source: Front Plant Sci. 2023 Jun 30;14:1201730. doi: 10.3389/fpls.2023.1201730 (PMC10347410; doi:10.3389/fpls.2023.1201730)
Supplement: Supplementary file 1 [file DataSheet_1.zip › Supplementary materiars/Figure S1. Three-dimensional domains of StSOS1s proteins.docx]

**
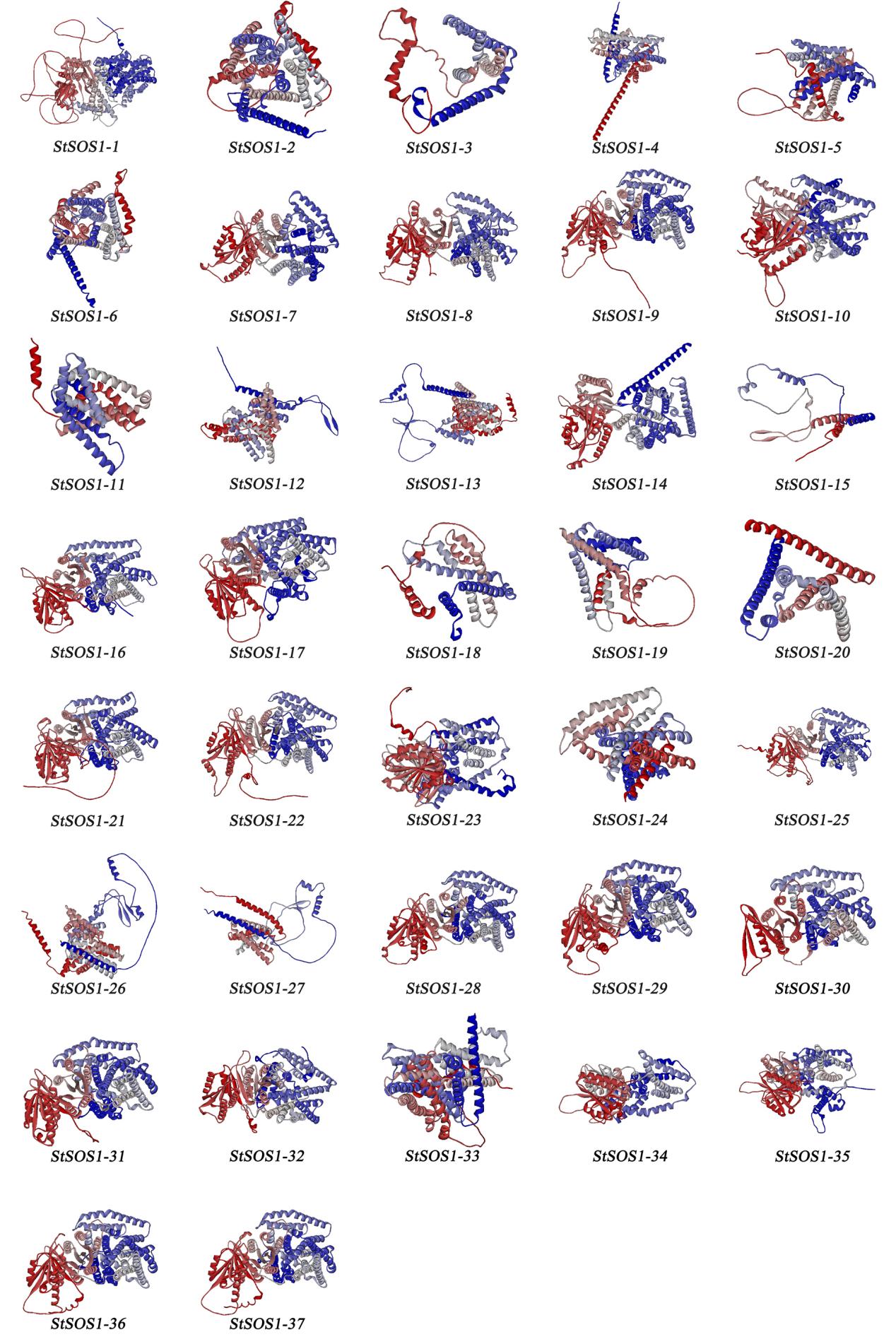
**

**Figure S1.** Comparison of the three-dimensional structures of StSOS1s proteins. The colored amino acid residues changes from blue to red from N-terminal to C-terminal sites. The structures were predicted for these proteins using AlphaFold protein structure database which utilizes artificial intelligence to predict a protein’s three-dimensional structure from the given amino acid sequence.
