# Supplementary material for: Genome-wide characterization of SOS1 gene family in potato (Solanum tuberosum) and expression analyses under salt and hormone stress
Source: Front Plant Sci. 2023 Jun 30;14:1201730. doi: 10.3389/fpls.2023.1201730 (PMC10347410; doi:10.3389/fpls.2023.1201730)
Supplement: Supplementary file 1 [file DataSheet_1.zip › Supplementary materiars/Figure S2. Interactions between proteins encoded by StSOS1s.docx]

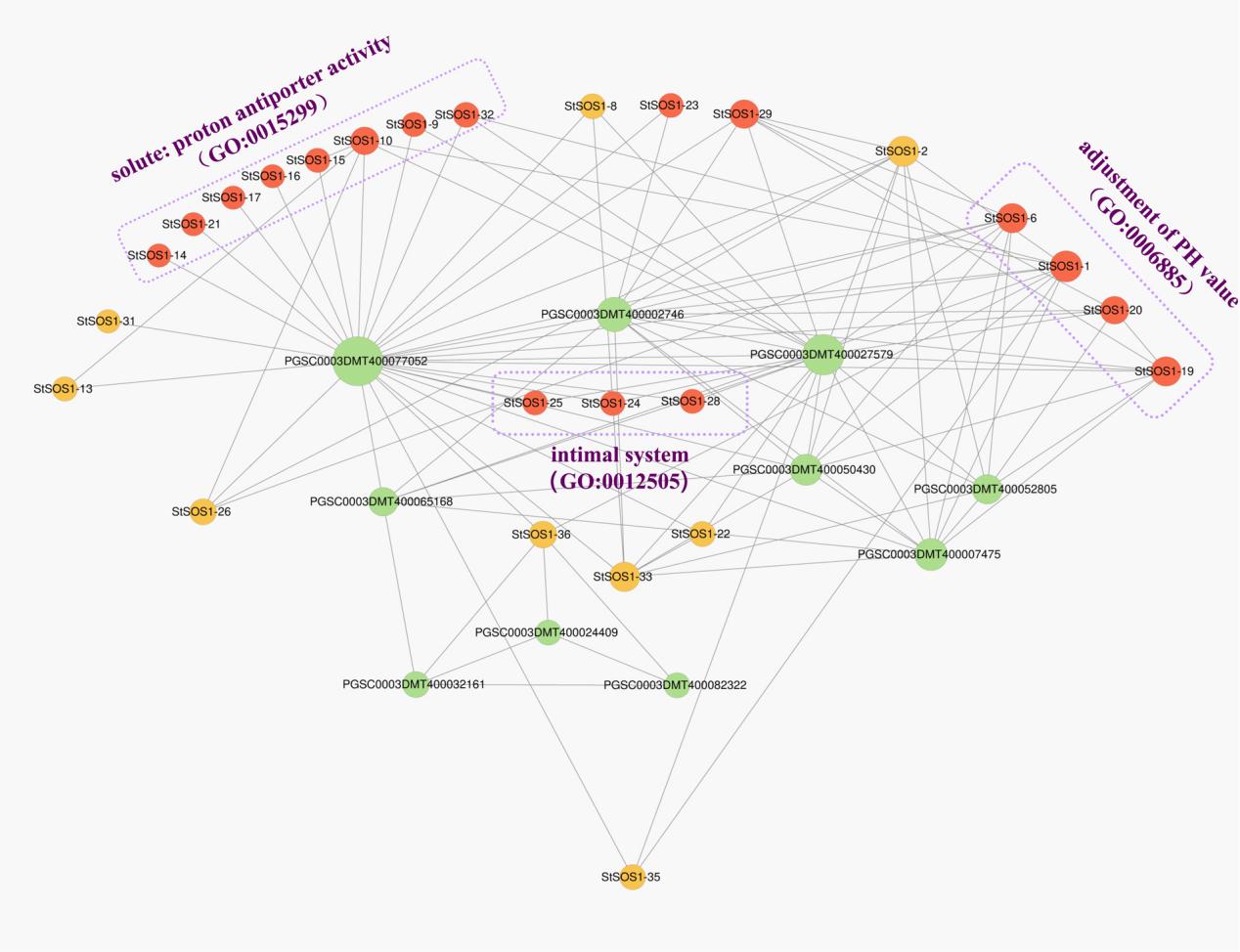


**Figure S2.** Protein-protein interaction network for all StSOS1s in potato determined using Cytoscape.

Dots represent nodes (or, proteins) and lines represent edges (or, connections among nodes). Edges represent protein-protein associations and the size of the circle indicates its importance in the network.


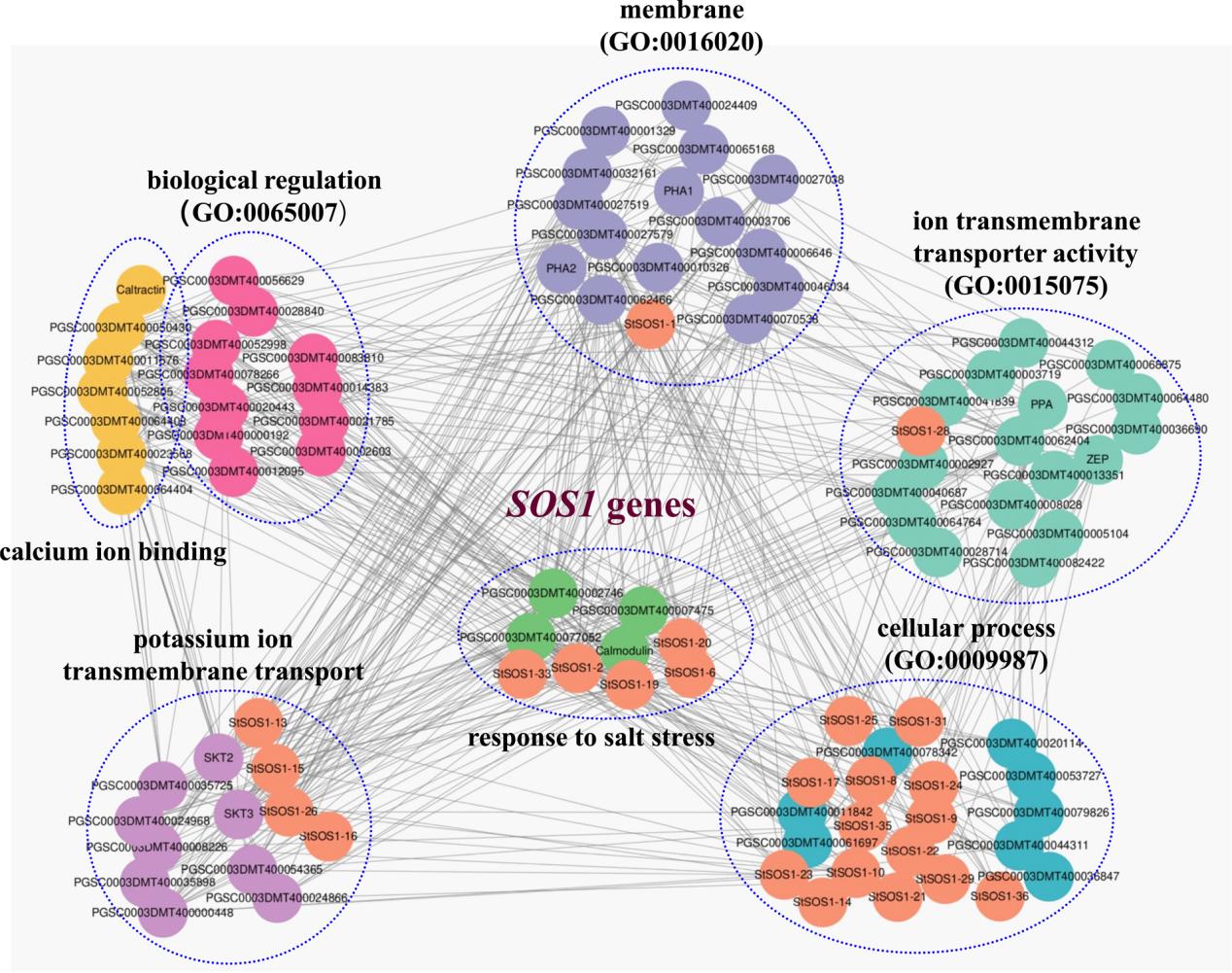


**Figure S3.** Protein-protein interaction network for 96 StSOS1s determined using Cytoscape.

107 protein-protein interactions are shown. Dots represent nodes (or, proteins) and lines represent edges (or, connections among nodes). The different colors represent the cluster genes with different functions classified with GO terms marked with dashed circles.
