# Supplementary material for: Genome-wide characterization of SOS1 gene family in potato (Solanum tuberosum) and expression analyses under salt and hormone stress
Source: Front Plant Sci. 2023 Jun 30;14:1201730. doi: 10.3389/fpls.2023.1201730 (PMC10347410; doi:10.3389/fpls.2023.1201730)
Supplement: Supplementary file 1 [file DataSheet_1.zip › Supplementary materiars/Table S1. Primers of qRT-PCR for StSOS1s genes.docx]

**Table S1.** Primers of qRT-PCR for *StSOS1* genes

| **No.** | **Prime name** | **Sense sequence (5'to3')** | **Reverse sequence (5'to3')** |
| --- | --- | --- | --- |
| 1 | *Actin* | TATAACGAGCTTCGTGTTGCAC | ACTGGCATACAGCGAAAGAACA |
| 2 | *StSOS1-1* | GGATTGGCGATGGTATCCGT | CAATCTTGAGGGCAGCTCCA |
| 3 | *StSOS1-2* | TGTAAGCGACAGCCCACAAT | GAGTATGGCCTCCCCTGGTA |
| 4 | *StSOS1-6* | CTCTTGGAGGAGAACCGCTG | GGTACCAATGGCTCCGAACA |
| 5 | *StSOS1-13* | TCCTGGAGACGGTAGCCAAA | ATTCCACCAATGGCAGCAGA |
| 6 | *StSOS1-28* | ACAACCCGAGGCGAACTTAG | ACACTTGTCCCGGAAACCTC |
| 7 | *StSOS1-29* | CTCTAGGCCGCAGTGAGAAG | GGCCACTCCCATAAACACCA |
