# Supplementary material for: Genome-wide characterization of SOS1 gene family in potato (Solanum tuberosum) and expression analyses under salt and hormone stress
Source: Front Plant Sci. 2023 Jun 30;14:1201730. doi: 10.3389/fpls.2023.1201730 (PMC10347410; doi:10.3389/fpls.2023.1201730)
Supplement: Supplementary file 1 [file DataSheet_1.zip › Supplementary materiars/Table S2. Number of identified phosphorylation sites in the StSOS1 proteins.docx]

**Table S2.** Number of identified phosphorylation sites in the StSOS1 proteins

|  | **Gene ID** | **Transcript ID** | **Phosphorylation site** |
| --- | --- | --- | --- |
| *StSOS1-1* | PGSC0003DMG400022786 | PGSC0003DMT400058653 | Ser:72, Thr:32, Tyr:7 |
| *StSOS1-2* | PGSC0003DMG400010663 | PGSC0003DMT400027658 | Ser:34, Thr:16, Tyr:3 |
| *StSOS1-3* | PGSC0003DMG400010663 | PGSC0003DMT400027657 | Ser:15, Thr:9, Tyr:2 |
| *StSOS1-4* | PGSC0003DMG400010663 | PGSC0003DMT400027656 | Ser:30, Thr:10, Tyr:2 |
| *StSOS1-5* | PGSC0003DMG400022490 | PGSC0003DMT400057914 | Ser:22, Thr:14, Tyr:2 |
| *StSOS1-6* | PGSC0003DMG400022490 | PGSC0003DMT400057913 | Ser:30, Thr:17, Tyr:2 |
| *StSOS1-7* | PGSC0003DMG400021928 | PGSC0003DMT400056443 | Ser:48, Thr:24, Tyr:7 |
| *StSOS1-8* | PGSC0003DMG400021928 | PGSC0003DMT400056445 | Ser:55, Thr:26, Tyr:7 |
| *StSOS1-9* | PGSC0003DMG400009710 | PGSC0003DMT400025130 | Ser:51, Thr:21, Tyr:3 |
| *StSOS1-10* | PGSC0003DMG400018689 | PGSC0003DMT400048101 | Ser:37, Thr:19, Tyr:5 |
| *StSOS1-11* | PGSC0003DMG400031029 | PGSC0003DMT400079669 | Ser:12, Thr:6, Tyr:1 |
| *StSOS1-12* | PGSC0003DMG400031029 | PGSC0003DMT400079670 | Ser:22, Thr:13, Tyr:2 |
| *StSOS1-13* | PGSC0003DMG400031029 | PGSC0003DMT400079671 | Ser:26, Thr:16, Tyr:2 |
| *StSOS1-14* | PGSC0003DMG400027255 | PGSC0003DMT400070102 | Ser:38, Thr:18, Tyr:2 |
| *StSOS1-15* | PGSC0003DMG400009808 | PGSC0003DMT400025403 | Ser:12,Thr:5, Tyr:0 |
| *StSOS1-16* | PGSC0003DMG400011649 | PGSC0003DMT400030419 | Ser:42, Thr:13, Tyr:5 |
| *StSOS1-17* | PGSC0003DMG400007292 | PGSC0003DMT400018809 | Ser:55, Thr:14, Tyr:6 |
| *StSOS1-18* | PGSC0003DMG402021988 | PGSC0003DMT400056557 | Ser:13, Thr:7, Tyr:3 |
| *StSOS1-19* | PGSC0003DMG402021988 | PGSC0003DMT400056556 | Ser:15, Thr:8, Tyr:3 |
| *StSOS1-20* | PGSC0003DMG402021988 | PGSC0003DMT400056555 | Ser:12, Thr:7, Tyr:0 |
| *StSOS1-21* | PGSC0003DMG402021988 | PGSC0003DMT400061554 | Ser:30, Thr:22, Tyr:9 |
| *StSOS1-22* | PGSC0003DMG400013814 | PGSC0003DMT400035881 | Ser:32, Thr:27, Tyr:1 |
| *StSOS1-23* | PGSC0003DMG400030375 | PGSC0003DMT400078102 | Ser:45, Thr:15, Tyr:2 |
| *StSOS1-24* | PGSC0003DMG400035252 | PGSC0003DMT400085681 | Ser:11, Thr:8, Tyr:2 |
| *StSOS1-25* | PGSC0003DMG400030154 | PGSC0003DMT400077544 | Ser:41, Thr:28, Tyr:6 |
| *StSOS1-26* | PGSC0003DMG400029945 | PGSC0003DMT400076994 | Ser:37, Thr:15, Tyr:3 |
| *StSOS1-27* | PGSC0003DMG400029945 | PGSC0003DMT400076993 | Ser:20, Thr:12, Tyr:1 |
| *StSOS1-28* | PGSC0003DMG400012169 | PGSC0003DMT400031718 | Ser:43, Thr:17, Tyr:2 |
| *StSOS1-29* | PGSC0003DMG400012168 | PGSC0003DMT400031717 | Ser:45, Thr:22, Tyr:4 |
| *StSOS1-30* | PGSC0003DMG400008849 | PGSC0003DMT400022808 | Ser:29, Thr:16, Tyr:3 |
| *StSOS1-31* | PGSC0003DMG400008849 | PGSC0003DMT400022809 | Ser:38, Thr:17, Tyr:3 |
| *StSOS1-32* | PGSC0003DMG400004171 | PGSC0003DMT400010686 | Ser:37, Thr:17, Tyr:8 |
| *StSOS1-33* | PGSC0003DMG400034953 | PGSC0003DMT400085382 | Ser:35, Thr:18, Tyr:5 |
| *StSOS1-34* | PGSC0003DMG400014998 | PGSC0003DMT400038811 | Ser:31, Thr:14, Tyr:7 |
| *StSOS1-35* | PGSC0003DMG400014998 | PGSC0003DMT400038812 | Ser:40, Thr:19, Tyr:6 |
| *StSOS1-36* | PGSC0003DMG400005009 | PGSC0003DMT400012866 | Ser:43, Thr:16, Tyr:7 |
| *StSOS1-37* | PGSC0003DMG400005009 | PGSC0003DMT400012865 | Ser:43, Thr:16, Tyr:7 |

* Ser: Serine, Thr: Threonine, Tyr: Tyrosine.
