# Supplementary material for: Genome-wide characterization of SOS1 gene family in potato (Solanum tuberosum) and expression analyses under salt and hormone stress
Source: Front Plant Sci. 2023 Jun 30;14:1201730. doi: 10.3389/fpls.2023.1201730 (PMC10347410; doi:10.3389/fpls.2023.1201730)
Supplement: Supplementary file 1 [file DataSheet_1.zip › Supplementary materiars/Table S3. The MEME motifs of SOS1s family from potato.docx]

**Table S3.** Identificaton of consensus sequence of *StSOS1s* using MEME and the functional sites in motifs by Eukaryotic Linear Motif resource programs (ELM). The motif numbers are the same to those described in Fig. 5.

| **Motif Number** | **size (aa)** | **E-value** | **Best possible match**  **(amino acid LOGO)** | **Functional sites in Motifs** | | |
| --- | --- | --- | --- | --- | --- | --- |
|  |  |  |  | **Positions** | **Cell Compartment** | **ELM Description** |
| Motif 1 | 50 | 6.4e-363 | 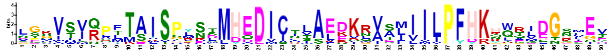  LGKVSVQPFTAISPYSTMHEDICTLAEDKRVSLIILPFHKHQRIDGSMEV | 7-15 | protein kinase CK2 complexl | Casein kinase 2 (CK2) phosphorylation site |
| Motif 2 | 50 | 1.2e-348 | 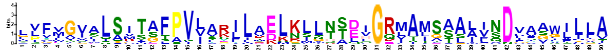  LVFVGVALSITAFPVLARJLAELKLLNSEIGRMAMSAALINDVAAWJLLA | 8-34 | centralspindlin complex, gamma-tubulin complex, midbody | Ser/Thr residue phosphorylated by the Plk1 kinase |
| Motif 3 | 35 | 2.4e-281 | 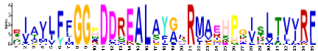  RIAVLFFGGPDDREALAYGKRMAEHPGISLTVIRF | 30-35 | SCF ubiquitin ligase complex | Ser/Thr residue phosphorylated by Plk4 |
| Motif 4 | 50 | 5.5e-257 | 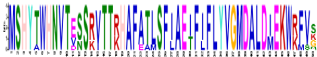  MSHYTWHNVTESSRVTTRHAFATLSFIAEIFIFLYVGMDALDIEKWRFVS | 5-9, 29-34, 44-49 | cytosol, cytoplasmic side of late endosome membrane | motif that binds to Atg8 protein family members to mediate processes involved in autophagy |
| Motif 5 | 50 | 5.5e-229 | 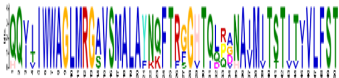  QQIIIWWAGLMRGAVSMALAYNQFTRGGHTQLPANAIMITSTITIVLFST | 21-24 | plasma membrane,  clathrin-coated endocytic vesicle,  cytosol | Tyrosine-based sorting signal responsible for the interaction with mu subunit of AP (Adaptor Protein) complex |
| Motif 6 | 21 | 5.4e-187 | 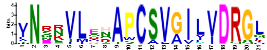  VNRRVLEHAPCSVGILVDRGL | 3-5 | extracellular,  Golgi apparatus | Yeast kexin 2 cleavage site (K-R-\|-X or R-R-\|-X).  N-Arg dibasic convertase (NRD/Nardilysin) cleavage site (X-\|-R-K or R-\|-R-X). |
| Motif 7 | 31 | 1.0e-173 | 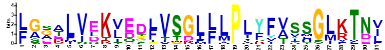  LGSALVEKVEDLVSGLLLPLYFVSSGLKTNV | 14-20 | cytosol,  internal side of plasma membrane | motif that binds to Atg8 protein family members to mediate processes involved in autophagy |
| Motif 8 | 29 | 2.0e-156 | 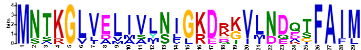  MNTKGLVELIVLNIGKDRKVLNDZTFAIM | 1-11 | nucleus, cytosol, Transcription factor complex | A kinase docking motif that mediates interaction towards the ERK1/2 and p38 subfamilies of MAP kinases |
| Motif 9 | 41 | 4.2e-197 | 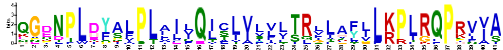  QGDNPLDYALPLAILQJCLVLVLTRLLAYJLKPLRQPRVVA | 2-28 | nucleus | nuclear receptor box motif (LXXLL) confers binding to nuclear receptors |
| Motif 10 | 50 | 5.3e-287 | 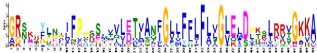  GRSKGYLNAJFPPSSLVVLETVANFGLLFFLFLVGLELDPKSLRRVGKKA | 4-21, 9-46 | cytosol, nucleus | GSK3 phosphorylation recognition site |

* ELM.http://elm.eu.org.

** MEME. https://meme-suite.org/meme
